# Long non-coding RNAs as novel therapeutic targets in juvenile myelomonocytic leukemia

**DOI:** 10.1038/s41598-021-82509-5

**Published:** 2021-02-02

**Authors:** Mattias Hofmans, Tim Lammens, Barbara Depreter, Ying Wu, Miriam Erlacher, Aurélie Caye, Hélène Cavé, Christian Flotho, Valerie de Haas, Charlotte M. Niemeyer, Jan Stary, Filip Van Nieuwerburgh, Dieter Deforce, Wouter Van Loocke, Pieter Van Vlierberghe, Jan Philippé, Barbara De Moerloose

**Affiliations:** 1 Department of Pediatric Hematology-Oncology and Stem Cell Transplantation, Ghent University Hospital, Ghent, Belgium; 2 Department of Diagnostic Sciences, Ghent University Hospital, Corneel Heymanslaan 10, Ghent, 9000 Belgium; 3 Cancer Research Institute Ghent, Ghent University, Ghent, Belgium; 4 Department of Laboratory Medicine Hematology, University Hospital Brussels, Brussels, Belgium; 5 Faculty of Biology, University of Freiburg, Freiburg, Germany; 6 Division of Pediatric Hematology and Oncology, Department of Pediatrics and Adolescent Medicine, University Medical Center Freiburg, Faculty of Medicine, University of Freiburg, Freiburg, Germany; 7 German Cancer Consortium, Partner Site Freiburg, German Cancer Research Center, Heidelberg, Germany; 8 Department of Genetics, University Hospital of Robert Debré (APHP) and INSERM U1131, Institut de Recherche Saint-Louis, Université de Paris, Paris, France; 9 Princess Máxima Center for Pediatric Oncology, Utrecht, The Netherlands; 10 Dutch Childhood Oncology Group, The Hague, The Netherlands; 11 Department of Pediatric Hematology/Oncology, Charles University and University Hospital Motol, Prague, Czech Republic; 12 Laboratory for Pharmaceutical Biotechnology, Faculty of Pharmaceutical Sciences, Ghent University, Ghent, Belgium; 13 Department of Biomolecular Medicine, Ghent University, Ghent, Belgium

**Keywords:** Myelodysplastic syndrome, Cancer genomics

## Abstract

Juvenile myelomonocytic leukemia (JMML) treatment primarily relies on hematopoietic stem cell transplantation and results in long-term overall survival of 50–60%, demonstrating a need to develop novel treatments. Dysregulation of the non-coding RNA transcriptome has been demonstrated before in this rare and unique disorder of early childhood. In this study, we investigated the therapeutic potential of targeting overexpressed long non-coding RNAs (lncRNAs) in JMML. Total RNA sequencing of bone marrow and peripheral blood mononuclear cell preparations from 19 untreated JMML patients and three healthy children revealed 185 differentially expressed lncRNA genes (131 up- and 54 downregulated). LNA GapmeRs were designed for 10 overexpressed and validated lncRNAs. Molecular knockdown (≥ 70% compared to mock control) after 24 h of incubation was observed with two or more independent GapmeRs in 6 of them. For three lncRNAs (*lnc-THADA-4*, *lnc-ACOT9-1* and *NRIR*) knockdown resulted in a significant decrease of cell viability after 72 h of incubation in primary cultures of JMML mononuclear cells, respectively. Importantly, the extent of cellular damage correlated with the expression level of the lncRNA of interest. In conclusion, we demonstrated in primary JMML cell cultures that knockdown of overexpressed lncRNAs such as *lnc-THADA-4*, *lnc-ACOT9-1* and *NRIR* may be a feasible therapeutic strategy.

## Introduction

Currently, hematopoietic stem cell transplantation (HSCT) is the established standard of care in juvenile myelomonocytic leukemia (JMML), a distinct myelodysplastic/myeloproliferative disorder of early childhood^1^. HSCT results in long-term overall survival (OS) of approximately 50–60% of patients^2^. Characteristic for JMML are proliferative features, such as monocytosis, splenomegaly, and a moderately elevated proportion of myeloblasts. These disease features are caused by clonal growth of an abnormal multipotent hematopoietic stem cell^3,4^. In approximately 90–95% of patients, canonical mutations in the *NRAS* and *KRAS* (20–25%), *PTPN11* (35%), *NF1* (10–15%) or *CBL* (10–15%) genes are observed, strongly linking the disorder to hyperactivation of the RAS/MAPK pathway. More recently, investigations of secondary mutations^5,6^, non-coding RNA expression^7,8^, and genomic DNA methylation^9–11^ have led to a better understanding of the pathobiology of the disease. The acquisition of secondary mutations and aberrant DNA methylation were found to be associated with unfavorable clinical characteristics and poor prognosis, and might be used in the future to guide treatment decisions^5,12^. Furthermore, methylome studies encouraged the use of the DNA methyltransferase-inhibiting agent azacitidine as a bridge to HSCT, with promising results in single case reports and the first clinical trial^13–16^.

Long non-coding RNAs (lncRNAs) are RNA transcripts with a minimum length of 200 nucleotides and little to no evidence of protein-coding capacity. LncRNAs have emerged as an additional layer of gene expression regulation and were shown to play a role in normal and malignant hematopoiesis^17,18^. Dysregulation of key lncRNAs involved in processes such as maintenance and differentiation of hematopoietic stem cells can result in the development of hematological malignancies^19^. Interestingly, compared to protein-coding messenger RNAs (mRNAs), lncRNAs generally have a more tissue or cell-type specific expression pattern, making them ideal candidates for targeted therapies^20,21^.

Recently, we documented the lncRNA landscape of JMML using microarray profiles from 44 untreated JMML patients and 7 healthy controls, and correlated lncRNA expression with clinical and molecular characteristics^7^. In addition, we discovered a subset of lncRNAs specifically overexpressed in JMML patients that could potentially serve as new therapeutic targets^7^.

GapmeRs, potent antisense oligonucleotides (ASOs), represent a unique tool to perturbate lncRNA expression, even more efficiently than RNA interference^22^. LNA GapmeRs were shown earlier to act in hematological and solid tumors. For example, we have shown that silencing of lncRNA *lnc-RTN4R-*1 induces cell death in ETV6-RUNX1 childhood B-cell precursor leukemia^23^. Likewise, Leucci et al. recently illustrated that silencing of the lncRNA *SAMMSON*, in Patient-Derived Tumor Xenograft models of melanoma, strongly decreases melanoma cell survival^24^.

Here, we further refined the lncRNA transcriptome in JMML patients using RNA sequencing (RNA-seq) and quantitative reverse transcriptase PCR (qPCR) and evaluated GapmeR ASOs as a therapeutic strategy targeting overexpressed lncRNAs in JMML*.*

## Results

### Patients characteristics

To identify differentially expressed lncRNAs in JMML, we profiled bone marrow (BM) (n = 15) or peripheral blood (PB) (n = 4) mononuclear cell preparations (MNCs) from 19 JMML samples and 3 BM samples from age-matched pediatric haematological normal patients (PNs) using total RNA-seq. The patient cohort consisted of 8 *PTPN11*, 4 *KRAS*, 5 *NRAS* and 2 *NF1* mutated patients. Karyotypic abnormalities were detected in 6/19 patients, including monosomy 7 in four cases. The median age at diagnosis was 26 months (range 1–159 months). Seventeen out of 19 patients (89.5%) received HSCT. Relapse after HSCT occurred in 4/17 patients and one patient died of transplant-related toxicity. Detailed patient characteristics are summarized in Supplemental Table S1.

### Differential RNA expression between JMML patients and normal bone marrow controls

In total, 167,569 genes were identified of which 58,096 corresponded to known Ensembl genes. Of these, 19,979 were coding, and 38,117 non-coding genes. Using edgeR, we identified 227 and 419 differentially expressed coding and non-coding genes, respectively.

**Figure 1:**
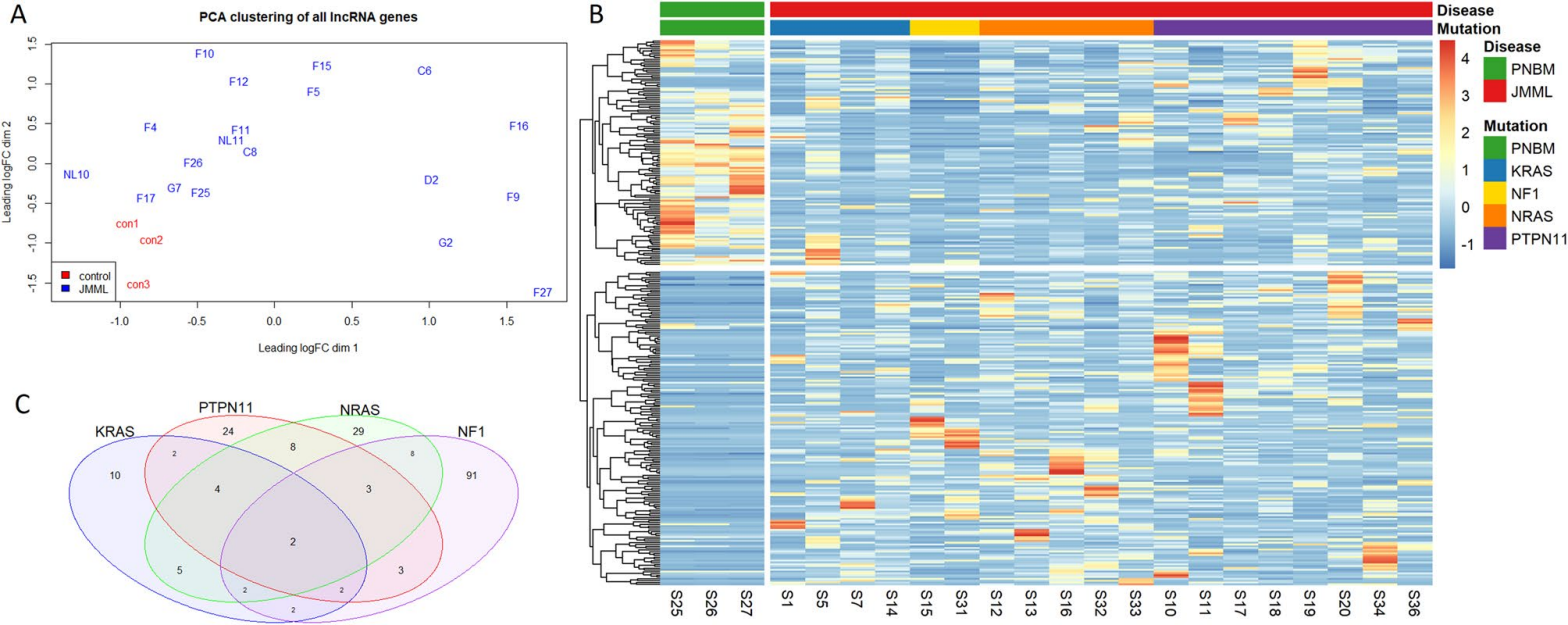
RNA sequencing reveals a deregulated lncRNA transcriptome in JMML patients. (**A**) Principal component analysis (PCA) clustering using normalized expression profiles of all known lncRNA genes (n = 8559) with expression of ≥ 1 count per million (CPM) in at least 2/3 of the patients or PN samples. JMML samples are depicted in red and PN BM in blue. Samples are depicted with their ID. (**B**) Expression heatmap of all lncRNAs deregulated in at least one JMML molecular subtype compared to controls (expression given as row scaled values). Differential expression analysis using EdgeR (R bioconductor) demonstrated presence of 281 significantly differentially expressed lncRNAs in at least 1 molecular subgroup compared to PN (adj. P ≤ 0.05). (**C**) Venn diagram showing the overlap of differentially expressed lncRNAs between JMML molecular subgroups and PNBM. *JMML* juvenile myelomonocytic leukemia, *PNBM* pediatric normal bone marrow*, lncRNA* long non-coding RNA*, RNA-seq* RNA sequencing, *PCA* principal component analysis.

The LNCipedia database was used to further identify 8559 known lncRNAs showing expression of ≥ 1 count per million in at least 2/3 of the patients or PN BM samples^21^. Principal component analysis (PCA), using this subset of lncRNAs showed clustering of PN BM samples and divergent of JMML patients (Fig. 1A). Within the JMML samples, heterogeneity can be observed. Moreover, a lncRNA signature was established, consisting of 185 differentially expressed lncRNAs genes (131 up- and 54 downregulated) between JMML and PN BM (Fig. 1B, Supplemental Table S2). In silico analysis with pre-ranked gene set enrichment analysis and lncRNA pathway analysis (see Supplementary methods) identified pathways associated with JAK-STAT-MAPK signaling, cancer development, and systemic inflammation to be synergistically regulated by these differentially expressed lncRNAs in JMML patients.

Pairwise analyses between *PTPN11* (n = 8), *KRAS* (n = 4), *NRAS* (n = 5) and *NF1* (n = 2) patients and PN were subsequently performed. Although these subgroups are relatively small, these analyses revealed 29 (19 up- and 10 downregulated) lncRNAs to be deregulated in *KRAS* mutated patients, 61 (49 up- and 12 downregulated) in *NRAS*, 48 (32 up- and 16 downregulated) in *PTPN11,* and 113 (29 up- and 84 downregulated) in *NF1* mutated patients compared to PN (Fig. 1C). Only *lnc-ACOT9-1* and *MKLN1-AS* were significantly upregulated in all mutational subgroups compared to PN samples, making them potential therapeutic targets. Six lncRNAs (five upregulated: *lnc-ACOT9-1*, *MKLN1-AS*, *lnc-BCAR1-1*, *lnc-SMG6-2* and *GUSBP11* and one downregulated: *SLC5A4-AS1*) showed differential expression in *PTPN11*, *NRAS* and *KRAS* mutated samples compared to PN samples. In total, 281 lncRNAs showed significantly differential expression in at least 1 molecular subgroup compared to PN (Supplemental Table S3). Only *lnc-KIFAP3-2*, showing upregulation in *PTPN11* vs *KRAS*, was found differentially expressed between *NRAS*, *KRAS* and *PTPN11* mutated patients, pointing to similar deregulated lncRNA profiles between these different molecular subgroups.

### Identification and validation of lncRNAs overexpressed in JMML

**Figure 2:**
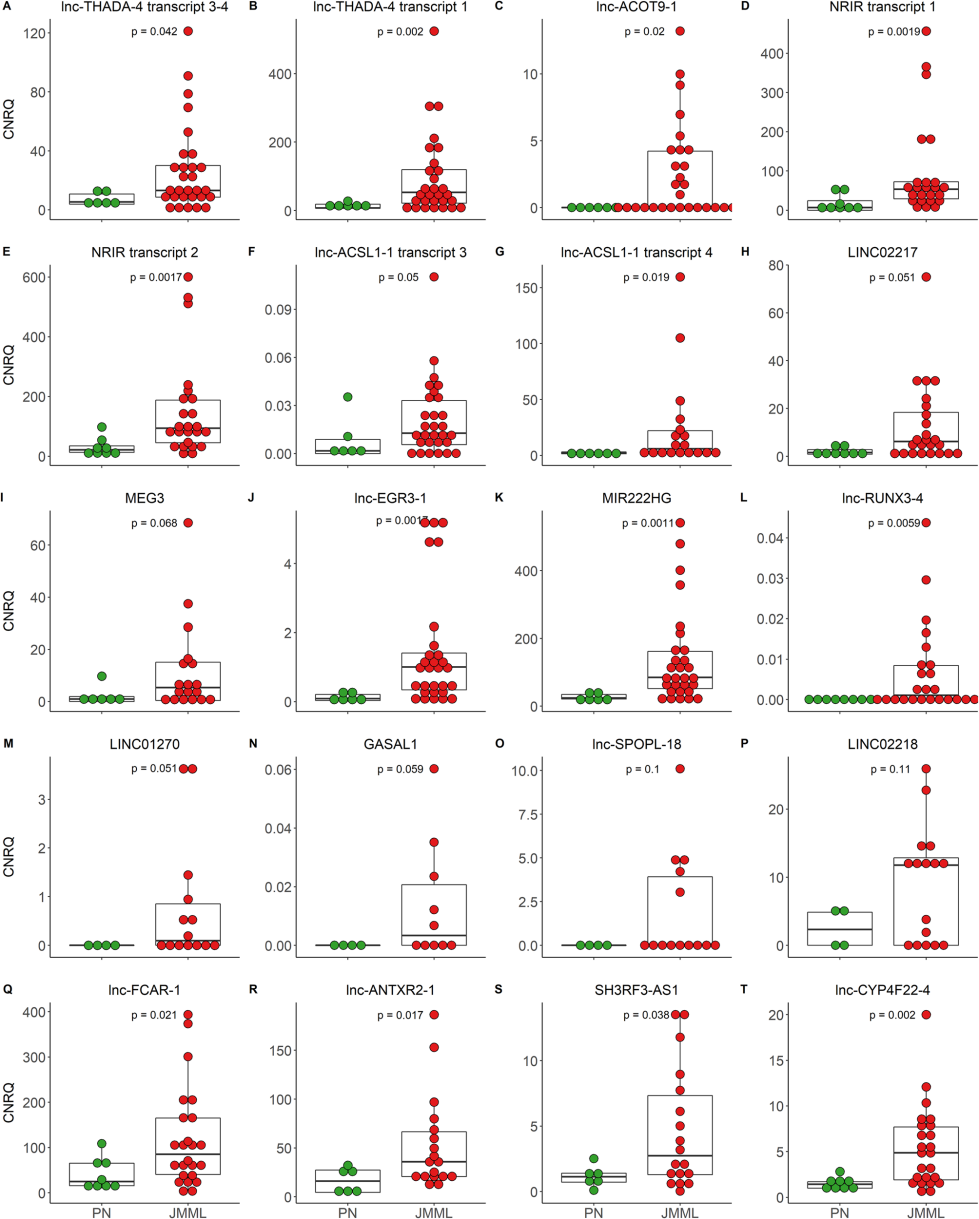
qPCR validation of the most significant differentially expressed lncRNAs discovered by RNA sequencing. qPCR confirmed significant upregulation of 12/45 (29%) and near significant upregulation of 6/45 (15%) lncRNA genes in JMML patients (n = 14 to 29) compared to PN (n = 6). One-sided P values were calculated by the Mann–Whitney U test. Median values and quartiles are shown by horizontal lines. The vertical axis (y-axis) corresponds to the calibrated normalized relative quantities (CNRQ). *TBP*, *HPRT1* and *GAPDH* were used as internal reference genes. *qPCR* quantitative reverse transcriptase PCR, *JMML* juvenile myelomonocytic leukemia, *PN* pediatric normal, *lncRNA* long non-coding RNA, *CNRQ* calibrated normalized relative quantity.

The top-25 overexpressed lncRNAs together with other lncRNAs, based on log2 fold change (logFC) and previously discovered differential expression^7^, were selected for validation with quantitative reverse-transcriptase polymerase chain reaction (qPCR). In total, in house designed and validated primers were available for 45 lncRNAs (Supplemental Table S4). In our independent validation cohort (12 JMML patients and 6 PN), significant and near-significant (P ≤ 0.1) overexpression was confirmed for 10 and 4 lncRNAs respectively (31%) (Supplemental Figure S1). However, due to the small sample size of this cohort, not all molecular subgroups were represented in sufficient numbers. After expansion of the validation cohort with patients from the discovery cohort, more lncRNAs showed significant or near significant overexpression (12 [29%] and 6 [15%] lncRNAs respectively) (Fig. 2; Supplemental Table S5). For 16/18 validated lncRNAs, sufficient data was present to perform a correlation analysis between RNA-seq and qPCR, and for 10/16 lncRNAs a significant and positive correlation could be observed (Supplemental Figure S2). Although upregulation was noted for both *lnc-ACOT9-1* and *MKLN1-AS* in all mutational subgroups in our RNA-seq data, this was only validated for *lnc-ACOT9-1* in the qPCR validation.

Detailed analysis of our qPCR results indicated that *lnc-THADA-4* was low in all of the *KRAS* mutated samples from our cohort (P = 0.006), whereas *lnc-EGR3-1* was overexpressed (> 3 SD above the average expression in PN) in all *KRAS* and *NF1* mutated patients (P = 0.044), and *LINC02217* in *NRAS* mutated patients (P = 0.02) (Supplemental Figure S3). None of the lncRNAs validated was associated with outcome in our cohort. However, *MIR222HG* overexpression was significantly associated with normal HbF levels (P = 0.032), overexpression of *SH3RF3-AS1* with low platelet counts (P = 0.045), overexpression of *LINC02218* with high platelet counts (P = 0.037) and overexpression of *lnc-SPOPL-18* with low blast counts (P = 0.049) at diagnosis.

### lncRNA knockdown in hematopoietic cell lines

Next, LNA GapmeRs were designed for each lncRNA with validated overexpression in JMML, to study the therapeutic potential and biological functions. To minimize off-target effects of individual GapmeRs, we aimed to evaluate a minimum of four different GapmeRs per lncRNA. For 8 lncRNAs it was not possible to design four LNA GapmeRs of sufficient predicted quality. For the remaining 10 lncRNAs (*lnc-THADA-4, lnc-ACOT9-1, NRIR, lnc-ACSL1-1, lnc-EGR3-1, MIR222HG, LINC02217, lnc-CYP4F22-4, lnc-RUNX3-4* and *MEG3),* GapmeRs could be designed and subsequently tested.

Since no JMML cell lines are available to evaluate GapmeRs, we screened publicly available RNA-seq data from 934 human cancer cell lines from the Cancer Cell Line Encyclopedia (CCLE) to identify hematopoietic cell lines with overexpression of the lncRNAs validated. For all these lncRNAs, at least one cell line from another pediatric or adult hematopoietic malignancy was selected and expression verified with qPCR (Supplemental Figure S4; Supplemental Table S6).

After 24 h incubation of cells with individual GapmeRs at a concentration of 5 µM, expression of the targeted lncRNA was determined with qPCR and compared with cells treated with empty mock control. For six lncRNAs (*lnc-THADA-4, lnc-ACOT9-1, NRIR, MIR222HG, LINC02217,* and *MEG3*) a downregulation ≥ 70% of the level of expression of the targeted lncRNA for two or more GapmeRs could be observed (Supplemental Figure S5).

**Figure 3:**
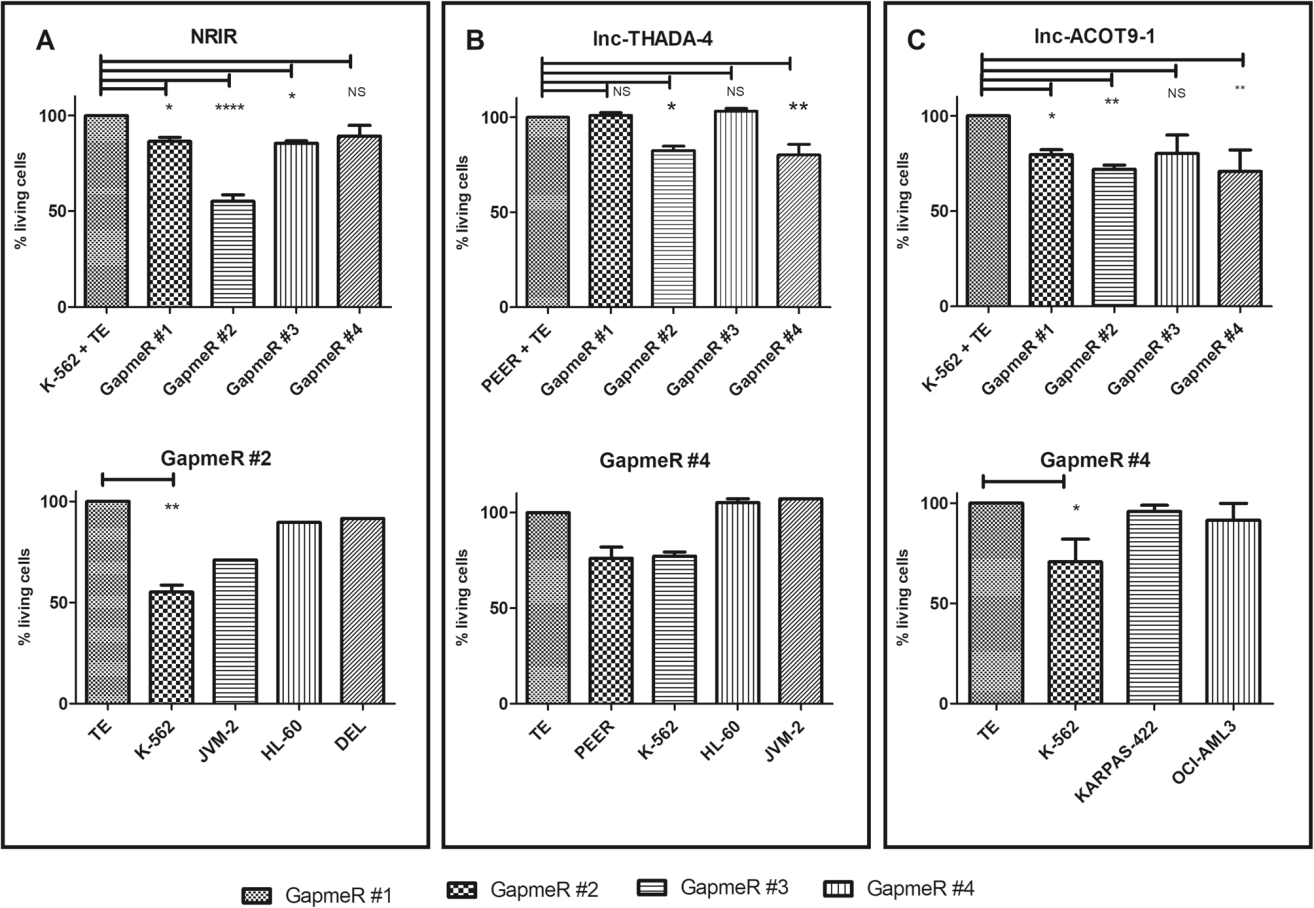
Effect of in vitro perturbation of lncRNAs using antisense LNA GapmeRs on cell viability of hematological cell lines. Data are shown for 3 different lncRNAs (*NRIR, lnc-THADA-4* and *lnc-ACOT9-1*) (**A**–**C**). Top: evaluation of cell death after treatment of different hematopoietic cell lines with highest expression of the lncRNA of interest with LNA GapmeRs (*NRIR* and *lnc-ACOT9-1* in K-562 and *lnc-THADA-4* in PEER). Evaluation by co-staining for annexin V and 7-AAD followed by flow cytometric analysis after 96 h of incubation with a concentration of 10 µM. The graphs are an average of three biological replicates and show the percentage of cells alive normalized against the mock control (TE-buffer). The percentage of remaining living cells is indicated on the y-axis ± SEM. Bottom: effect on cell death for the given LNA GapmeR in different hematopoietic cell lines with variable molecular expression of the lncRNA of interest. One-sided P values were calculated by one-way ANOVA with Bonferroni’s test and significance levels are indicated using asterisks. *P ≤ 0.05, **P ≤ 0.01, ****P ≤ 0.0001, *NS* not significant. *LNA* locked nucleic acid, *TE-buffer* tris–EDTA buffer, *SEM* standard error of the mean*, µM* micro molar.

Interestingly, treatment of PEER (childhood T acute lymphoblastic leukemia) with a GapmeR against *lnc-THADA-4* and treatment of K-562 (blast phase chronic myelogenous leukemia) with GapmeRs against *lnc-ACOT9-1* and *NRIR*, resulted in a significant reduction of cell viability after 96 h (− 20% for GapmeR #4 against *lnc-THADA-4* in PEER, − 45% for GapmeR #2 against *NRIR* in K-562, and − 30% for GapmeR #4 against *lnc-ACOT9-1* in K-562) (Fig. 3). Moreover, a correlation between the level of expression and % apoptotic cell death could be observed (R = 0.85, 0.9 and 0.46 for lnc-ACOT9-1, NRIR and lnc-THADA-4, respectively). Of note, off-target effects were unlikely as these GapmeRs did not show effect in hematopoietic cell lines without expression of the targeted lncRNA (Fig. 3).

### lncRNA knockdown and effect on cell viability in JMML patient cells in vitro

**Figure 4:**
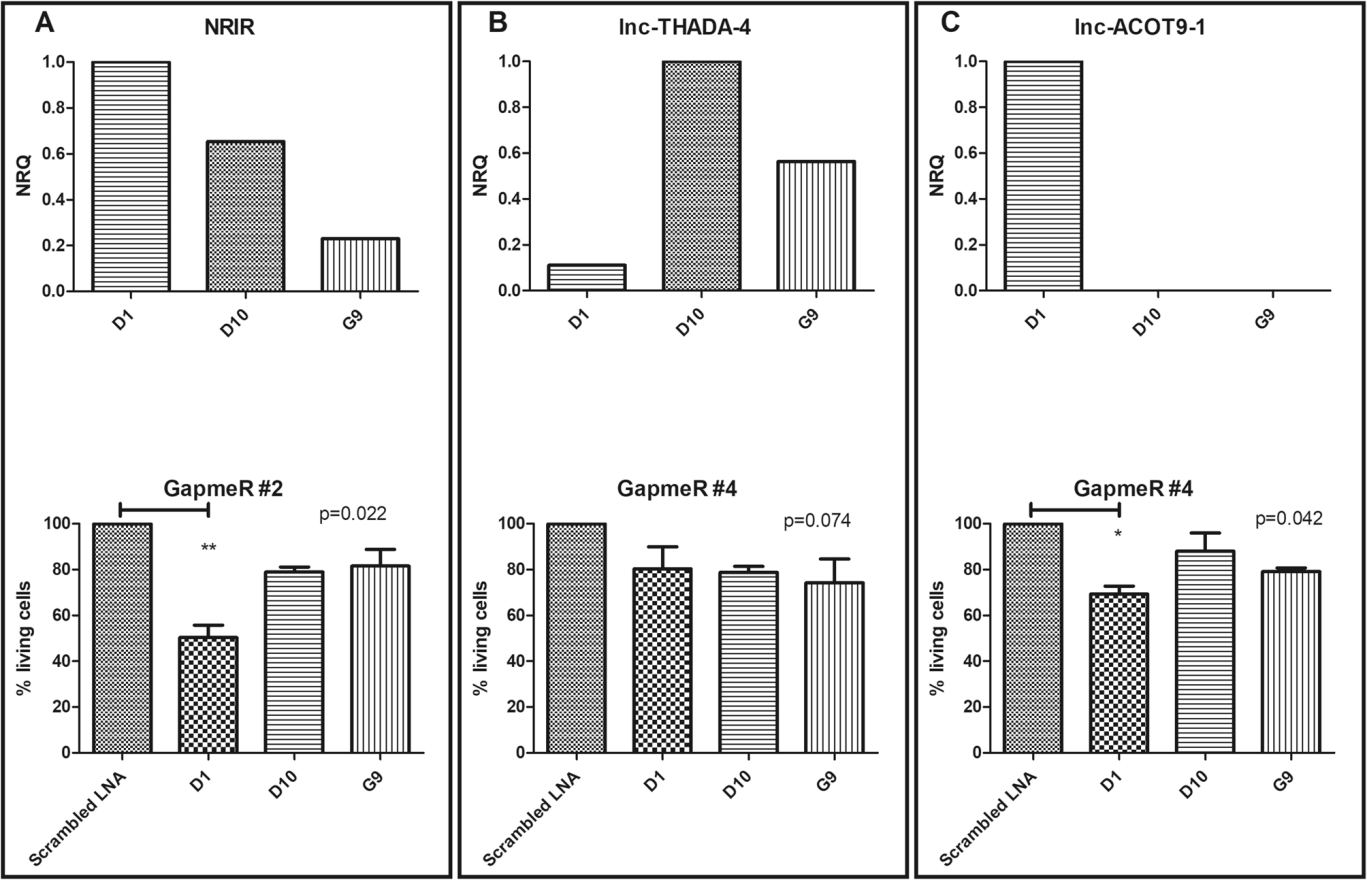
In vitro perturbation of lncRNAs in primary cultures of JMML MNCs. (**A**–**C**) GapmeRs against lncRNAs *NRIR, lnc-THADA-4* and *lnc-ACOT9-1* were in vitro evaluated in primary cultures of JMML MNCs (n = 3). Top: lncRNA expression was evaluated with qPCR in three primary cultures of JMML cells. Expression shown is relative to the average of three different housekeeping genes (*TBP*, *HPRT1* and *GAPDH*) and rescaled against the primary culture with the highest expression. Bottom: evaluation of cell death after treatment of primary cultures with LNA GapmeRs against *lnc-THADA-4*, *lnc-ACOT9-1* and *NRIR*. Evaluation was performed after 72 h of incubation with 10 µM by co-staining for annexin V and 7-AAD followed by flow cytometric analysis. The graphs are an average of three technical replicates and show on the y-axis the percentage of cells alive normalized against primary cultures treated with scrambled LNAs (mean ± SEM). One-sided P values were calculated by one-way ANOVA with Bonferroni’s test and significance levels are indicated using asterisks. *P ≤ 0.05, **P ≤ 0.01. *qPCR* quantitative reverse transcriptase PCR, *JMML* juvenile myelomonocytic leukemia, *MNCs* mononuclear cells*, lncRNA* long non-coding RNA, *SEM* standard error of the mean*, LNA* locked nucleic acid, *µM* micro molar.

As cell lines from other hematopoietic malignancies are no perfect disease models for JMML, we decided to study the effect of GapmeRs against *lnc-THADA-4*, *lnc-ACOT9-1* and *NRIR* in primary JMML cultures. To this end, MNCs from three JMML patients were cultured. Confirming our previous observations, *lnc-THADA-4* was highest expressed in the two *PTPN11* mutated patients (D10 and G9), whereas *lnc-ACOT9-1* was highest expressed in the KRAS mutated patient (D1). All three patients demonstrated variable expression of *NRIR* (Fig. 4A–C). Effect on cell death was subsequently evaluated after incubating the MNC cultures for 72 h with GapmeRs targeting *lnc-ACOT9-1, NRIR* and *lnc-THADA-4*. Treatment with GapmeR #4 targeting *lnc-ACOT9-1* resulted in a significant reduction of viable cells in patient D10 (− 30.7%), correlating with the level of expression (R = 0.88). Similarly, treatment with GapmeR #2 targeting *NRIR* also induced a significant reduction of viable cells in this patient (− 49.7%), correlating with *NRIR* expression (R = 0.87). GapmeRs targeting *lnc-THADA-4* caused a reduction of viability (− 21.3% and − 25.8% for D10 and G9, respectively) compared with a scrambled GapmeR, although not significant.

## Discussion

JMML is a very rare pediatric disease in which HSCT remains the main option for cure, but is associated with major morbidity. In addition, many patients still experience relapse. Thus, the identification of novel therapeutic targets is warranted. Consensus is growing that lncRNAs are involved in solid and hematopoietic human cancers and that cancer-associated lncRNAs might reveal new prognostic biomarkers. Moreover, using RNA-based therapies, cancer-associated lncRNAs may serve as specific therapeutic targets^25^. With this study, we demonstrate that lncRNA perturbation using LNA GapmeRs may serve as a novel therapeutic option in JMML in vitro.

Using total RNA-seq in a cohort of 19 JMML patients, we identified 185 differentially expressed lncRNAs. Despite the generally lower expression of lncRNAs, we were still able to validate approximately 45% of overexpressed lncRNAs with qPCR. Of note, 5/13 lncRNAs, i.e. *lnc-THADA-4*, previously identified as upregulated in JMML^7^, could be confirmed in this study. RNA profiles may differ between BM and PB samples, and both BM (n = 15) and PB (n = 4) samples were present in our RNA-seq cohort. Nevertheless, differential expression analyses between BM and PB samples did not reveal differentially expressed lncRNAs.

As JMML is a very rare disease it was not possible to establish an independent validation cohort of sufficient size for all molecular subgroups. However, when using 12 independent samples as validation cohort we found comparable although less powered results. In addition to the scarcity of diagnostic samples, no JMML specific cell lines are available for research. Therefore, it is still difficult to study novel therapies for this disease. We tackled this problem by using cell lines of other hematopoietic malignancies as surrogates and by generating primary JMML-cell cultures. We demonstrated that gymnotic delivery of LNA GapmeRs targeting overexpressed lncRNAs in JMML can decrease cell proliferation in both hematopoietic cell lines and primary MNC cultures of JMML patients. LNA GapmeRs against *lnc-THADA-4*, *NRIR* and *lnc-ACOT9-1* could impact cell viability with effects of – 30 to − 50%, 72–96 h after a single dose. Moreover, we could demonstrate a correlation between GapmeR effect and the level of molecular expression of the lncRNA of interest.

For most lncRNAs functional characterization is still lacking. It is known that some lncRNAs can regulate local chromatin structure and the expression of neighboring genes through its ability to recruit regulatory factors to the locus and/or modulate their function. Interestingly, *NRIR* is located close to the protein-coding gene *CMPK2*, which is overexpressed in chronic myeloid leukemia K-562 and lymphoblastic leukemia MOLT-4 cell lines, implicating a role in the pathophysiology of these diseases^26^. Our data demonstrated a high Spearman correlation coefficient between *NRIR* and *CMPK2* (p value = 0.002; R = 0.87). Other mRNA genes with high correlation with *NRIR* are alpha interferon-induced genes and *PKR* (protein kinase R; R = 0.90 and p value < 0.001). Increased expression of the latter gene has been reported in acute leukemia and solid tumors and correlates with worse survival and shortened remission duration in AML^27^. *Lnc-ACOT9-1* is surrounded by the protein-coding genes *SAT1* and *ACOT9*, both of which are overexpressed in JMML patients based on our previously generated microarray data. Noteworthy, *lnc-THADA-4* was found to be essential for cellular growth of chronic myeloid leukemia K-562 cells in a genome-wide screening for functional lncRNAs by Cas9 targeting of splice sites^28^. Our finding of decreased cell viability of overexpressing cell lines and primary culture of JMML cells after knockdown of *lnc-THADA-4* further corroborates the postulated essential role of this lncRNA.

Chemical modifications, like LNAs, could increase potency and simultaneously increase affinity to unintended targets with partial sequence complementarity^29^. As we did not observe a decrease in cell viability in cells without molecular expression of the lncRNA, against which the GapmeR is targeted, severe off-target effects seem improbable. GapmeRs are already employed for other diseases and are currently in phase 1 and 2 clinical trials for centronuclear myopathy. However, the safety and efficacy of the GapmeRs we designed in this study need to be validated in in vivo models. In particular, hepatotoxicity is being observed with some LNA-modified GapmeRs and thus requires extensive testing^30,31^. Recently, two in vivo models have been described and can be used for this purpose, one in Rag2^−/−^gamma(c)^−/−^ mice and one in NOD-scid IL2Rgnull-3/GM/SF (NSGS) mice^32–34^.

As only part of cells were killed, we propose to use sequential dosings or to combine different GapmeRs against key lncRNAs or combine GapmeRs with other treatment modalities (e.g. chemotherapy, hypomethylating agents, etc.) to increase killing efficacy of JMML cells both in vitro as in vivo. It is still difficult to study novel therapies in this rare disease, due to limited samples and the absence of JMML cell lines. Therefore, the generation of stable in vivo models as described above will be crucial.

In summary, our study further refines the lncRNA transcriptome in JMML and illustrates that lncRNA targeting through LNA GapmeRs could be a promising new approach in the treatment of JMML. We demonstrated in primary JMML cell cultures that knockdown of overexpressed lncRNAs such as l*nc-THADA-4*, *lnc-ACOT9-1* and *NRIR* may be a feasible therapeutic strategy.

## Methods

### Patient samples

Diagnostic BM (n = 15) and PB (n = 4) samples from 19 children with JMML and BM of 3 healthy children selected for sibling graft donation (PN) were used for RNA-seq. Sequencing results were verified with qPCR in a validation cohort consisting of 12 additional JMML patients (9 BM and 3 PB) and 6 PN BM samples. Additionally, living cells from 3 JMML patients, derived from spleen or PB, were used for primary cell culture generation. JMML samples were obtained from different institutions throughout Europe (Belgium, the Netherlands, France, the Czech Republic and Germany) (Supplemental Table S1). Out of 33 JMML patients, 17 are registered in the European Working Group of Myelodysplastic Syndromes in Childhood (EWOG-MDS) studies EWOG-MDS98 and EWOG-MDS2006 (National Institutes of Health trials registered as #NCT00047268 and #NCT00662090 at www.clinicaltrials.gov), and 16 in the French national JMML biobank. PN samples were collected at the Ghent University Hospital. Written informed consent was obtained from the parents of the patients or the healthy children before sample collection and approval for the study was granted from institutional review committees at each participating center. All experiments and methods were performed in accordance with the relevant guidelines and regulations.

### Cell lines and primary JMML cell culture

Hematopoietic cell lines PEER, K-562, MONO-MAC-6, HL-60, OCI-AML3, JURKAT, DAUDI, KASUMI-1, LOUCY, MV-4-11, and THP-1 were available in house, DEL and JVM-2 were purchased at the DSMZ repository (Braunschweig, Germany), and KARPAS-422 at Sigma-Aldrich (Saint Louis, Missouri, USA). Cell lines were grown in RPMI medium (Invitrogen, Waltham, MA, USA) supplemented with 10% or 20% Fetal Calf Serum (FCS, ThermoFisher Scientific, Waltham, MA, USA), according to supplier instructions, together with 100 U/mL Penicillin/Streptomycin (10,000 U/mL, Invitrogen) and 100 µg/mL l-glutamine (200 mM, Invitrogen). For THP-1, medium was additionally supplied with 0.05 mM β-mercaptoethanol. Cell lines were incubated at 37 °C in 5% CO_2_ incubators.

Mononuclear cell preparations derived from spleen and PB from 3 JMML patients were primary cultured in StemSpan SFEM II medium (Stemcell Technologies, Vancouver, Canada) supplemented with recombinant human IL3 (0.01 µg/mL; PeproTech, London, UK), FLT3L (0.01 µg/mL; PeproTech, London, UK), TPO (0.01 µg/mL; PeproTech, London, UK) and SCF (0.025 µg/mL; PeproTech, London, UK) and incubated at 37 °C in 5% CO_2_ incubators.

### RNA isolation, RNA sequencing, differential gene expression analysis, functional lncRNA analysis, complementary cDNA synthesis and quantitative reverse transcriptase PCR

Detailed information can be found in Supplementary Methods. Briefly, MNCs from JMML and PN BM were isolated and total RNA was extracted using TRIzol. Samples in the discovery cohort were used for total paired-end RNA-seq, whereas samples in the validation cohort were used for cDNA synthesis and qPCR.

### LncRNA perturbation

For each lncRNA of interest, a minimum of four antisense LNA GapmeRs (Qiagen) were designed using the LNA GapmeR designer (Qiagen). GapmeRs were resuspended in Tris–EDTA buffer and stored at − 20 °C. LncRNA expression of selected cell lines and primary cells was verified with qPCR. Cells from cell lines and MNCs from primary patients were plated in 96-well plates and incubated at 37 °C with different concentrations of GapmeRs (typically 1–10 µM) for 96 h and 72 h, respectively. Molecular efficiency of GapmeR treatment was assessed after 24 h with qPCR and knockdown was considered successful if downregulation of ≥ 70% of the lncRNA could be observed compared to the mock control. To test the passive uptake process (gymnosis) and nuclear migration in each sample, GapmeRs against the nuclear lncRNA *MALAT1* were used as positive control. Scrambled GapmeRs (Qiagen) were used as negative controls. The effect on cell viability was assessed using annexin-V and 7-AAD staining on a BD FACSCanto II flow cytometer (BD, San Jose, USA).

### Statistics

Graphics and statistical calculations were made in GraphPad Prism (version 5.04, La Jolla, CA, USA) and R bioconductor (version 3.11 ). Genes were considered differentially expressed if adjusted P value ≤ 0.05 and absolute log2 fold change (logFC) > 2. Unpaired Student’s T test or Mann–Whitney *U* test were used to compare continuous data between two groups. Pearson Chi-square test was used to compare categorical data. Statistical analyses involving more than two groups were performed by one-way analysis of variance followed by the Bonferroni post-test, or Kruskal–Wallis test followed by the Dunn test. Pearson correlation was performed to measure the degree of association between 2 continuously measured variables. P values > 0.05 were reported as non-significant, with those between 0.05 and 0.1 shown in detail. Data are expressed as the mean ± standard error of mean (SEM) of three independent experiments, unless otherwise indicated.

### Ethics approval

Approval for the study was granted from the institutional review committee at the Ghent university hospital (EC/2011/825) and at each participating center.

### Informed consent

Written informed consent was obtained from the parents of the patients or the healthy children before sample collection.

## Supplementary Information

Supplementary Information

## Data Availability

The datasets generated during and/or analysed during the current study are available in the National Center for Biotechnology Information (NCBI) Gene Expression Omnibus (GEO) database and are available at GEO (accession number GSE147523).
