# Supplementary material for: Long non-coding RNAs as novel therapeutic targets in juvenile myelomonocytic leukemia
Source: Sci Rep. 2021 Feb 2;11:2801. doi: 10.1038/s41598-021-82509-5 (PMC7854679; doi:10.1038/s41598-021-82509-5)

Long Non-Coding RNAs as Novel Therapeutic Targets in Juvenile Myelomonocytic Leukemia

Mattias Hofmans[1,2*], Tim Lammens[1,3], Barbara Depreter[4], Ying Wu[5,6], Miriam Erlacher[6,7], Aurélie Caye[8], Hélène Cavé[8], Christian Flotho[6,7], Valerie de Haas[9,10], Charlotte M. Niemeyer[6,7], Jan Stary[11], Filip Van Nieuwerburgh[12], Dieter Deforce[12], Wouter Van Loocke[13], Pieter Van Vlierberghe[3,13], Jan Philippé[2,3], Barbara De Moerloose[1,3]

1. Department of Pediatric Hematology-Oncology and Stem Cell Transplantation, Ghent University Hospital, Ghent, Belgium
2. Department of Diagnostic Sciences, Ghent University, Ghent, Belgium
3. Cancer Research Institute Ghent, Ghent University, Ghent, Belgium
4. Department of Laboratory Medicine Hematology, University Hospital Brussels, Brussels, Belgium
5. Faculty of Biology, University of Freiburg, Freiburg, Germany
6. Division of Pediatric Hematology and Oncology, Department of Pediatrics and Adolescent Medicine, University Medical Center Freiburg, Faculty of Medicine, University of Freiburg, Freiburg, Germany
7. German Cancer Consortium, partner site Freiburg, German Cancer Research Center, Heidelberg, Germany
8. Department of Genetics, University Hospital of Robert Debré (APHP) and INSERM U1131, Institut de Recherche Saint-Louis, Université de Paris, Paris, France
9. Princess Máxima Center for Pediatric Oncology, Utrecht, The Netherlands
10. Dutch Childhood Oncology Group, The Hague, The Netherlands
11. Department of Pediatric Hematology/ Oncology, Charles University and University Hospital Motol, Prague, Czech Republic
12. Laboratory for Pharmaceutical Biotechnology, Faculty of Pharmaceutical Sciences, Ghent University, Ghent, Belgium
13. Department of Biomolecular Medicine, Ghent University, Ghent, Belgium

***corresponding author:** Mattias Hofmans, Department of Pediatric Hematology-Oncology and Stem Cell Transplantation, Ghent University Hospital, Corneel Heymanslaan 10, 9000, Ghent, Belgium

e-mail: Mattias.hofmans@ugent.be

Supplementary Methods

RNA isolation, RNA sequencing and differential gene expression analysis

Mononuclear cells were isolated from all patients and PNBMs and total RNA was extracted in different labs from stored, frozen cells using Trizol. Also, RNA from a selection of haematopoietic cell lines was extracted using Trizol and the miRNeasy Mini Kit (Qiagen, Hilden, Germany) or the miRNeasy Micro Kit (Qiagen) in combination with on-column DNase I digestion (RNase-Free DNase set, Qiagen) according to manufacturer's instructions. The quality of the extracted RNA was assessed using the RNA 6000 Nano and RNA 6000 PICO assays on a 2100 Bioanalyzer (Agilent, Santa Clara, California, USA) and concentration was determined on a NanoDrop 2000/2000 c (ThermoFisher Scientific, Waltham, Massachusetts, USA).

RNA from 19 JMML patients and 3 PN BMs (= the discovery cohort) were used for paired-end total RNA sequencing. Library preparation and sequencing was performed by NXTGNT sequencing facility from Ghent University. Libraries were prepared using a TruSeq Stranded Total RNA Kit adapted for long fragments (+/- 550 bp) with the Ribo-Zero Gold rRNA Removal Kit (Illumina, San Diego, California, USA), according to the manufacturer's instructions. Prepared libraries were run on a HiSeq3000 high-throughput sequencing system (Illumina) and paired-end reads were generated (average of 52 million reads per sample). Obtained reads were aligned on the hg38 reference genome with STAR v2.4.2[1] and transcript assembly was perfomed with StringTie v1.3.3b.[2] Normalized counts were obtained using DESeq2. Briefly, raw count data for each gene obtained by RNA-seq were normalized by variance stabilizing transformation and the values were adjusted to center the genes relative to the medians. Differential expression analysis was performed with EdgeR (R Bioconductor, version 3.11).[3,4] Genes were considered differentially expressed if adjusted P-value ≤ 0.05 and absolute log2 fold change > 2. Clustering of samples was performed using t-distributed Stochastic Neighbor Embedding (t-SNE) of normalized counts. The data have been deposited in NCBI's Gene Expression Omnibus2 and are accessible through GEO Series accession number GSE147523.

Functional lncRNA analysis

As lncRNAs are less well characterized compared to mRNAs, it is difficult to predict their function. Therefore we used pre-ranked gene set enrichment analysis (GSEA). First, spearman's rho values (ρ value) were calculated between the specific lncRNAs of interest and all protein coding genes. This list was then used as input for preranked gene set enrichment analysis (GSEA) using GSEA software and the C2 Molecular Signature Database with 4729 curated gene sets (http://www.broad.mit.edu/gsea/) as previously described.[5] Secondly, an lncRNA-mRNA interaction network was used to functionally study differentially expressed lncRNAs (lncPath, R Bioconductor, version 3.11).[6] Hereby, pathways synergistically regulated by a set of lncRNAs of interest are identified based on a global network propagation method.

Complementary cDNA synthesis and quantitative real-time PCR

cDNA was synthesized for all JMML patients and PNBM samples from the validation cohort and for a selection of haematopoietic cell lines. cDNA synthesis was performed after an additional in-solution gDNase elimination step (Heat&Run gDNA removal kit, ArcticZymes, Tromsø, Norway), using the 5x PrimeScriptTM RT Master Mix (Takara Bio Europe SAS, Saint-Germain-en-Laye, France) in a final volume of 12.5 µL and diluted until a final concentration of 2.38 ng cDNA/µL.

qPCR reactions were carried out in 96-well plates using 0.3 µM primers, 2x Takyon Low ROX SYBR 2X MasterMix (Eurogentec, Liege, Belgium), 2.38 ng cDNA and H2O (Sigma-Aldrich, Saint Louis, Missouri, USA) in a 10 µL reaction. Samples were run in duplicate after a heat-activation step (3 min 95 °C) by a 2-step real-time protocol of 45 cycles (95 °C 15 sec, 60 °C 60 sec) on a Viia7 Real-Time PCR system (ThermoFisher Scientific, Waltham, Massachusetts, USA), combined with melting curve analysis (65 °C to 95 °C, gradually increasing with 0.5 °C/5 sec). Cq thresholds were automatically determined by the QuantStudio Real-Time PCR Software (ThermoFisher Scientific). Only results for which the difference between Cq values of the duplicates was below 0.5 for Cq < 30, below 1 for Cq > 30 and < 33, below 1.5 for Cq > 33 and < 36 and below 2 for Cq values > 36 were accepted and retained for statistical

analysis. LncRNA primers were in house designed and purchased at IDT Technologies (Coralville, Iowa, USA) (Supplementary table 2). *GAPD*, *HPRT1* and *TBP* were used as housekeeping genes and *MALAT1* was used to check for efficient LNA GapmeR knockdown trough gymnosis in the selected haematopoietic cell lines or primary culture of JMML cells. A generalized model of the delta-delta-Ct approach was used for statistical analysis. This method was based on Hellemans, J. et al.[7] Cq values generated for each target were corrected for primer pair efficiency and expressed as relative quantities (RQ). Normalised relative quantities (NRQ) were calculated by normalising RQ values against the expression of housekeeping genes *GAPD*, *HPRT1* and *TBP*. To allow inter-run comparison, calibrated NRQ values (CNRQ) were generated by taking into account the expression of a single inter-run calibrator (IRC), evaluated in each run by the respective primer pair.[7]

References

1 Dobin, A. *et al.* in *Bioinformatics* Vol. 29 15-21 (2013).
2 Pertea, M. *et al.* StringTie enables improved reconstruction of a transcriptome from RNA-seq reads. *Nature biotechnology* **33**, 290-295, doi:10.1038/nbt.3122 (2015).
3 Robinson, M. D., McCarthy, D. J. & Smyth, G. K. in *Bioinformatics* Vol. 26 139-140 (2010).
4 McCarthy, D. J., Chen, Y. & Smyth, G. K. in *Nucleic acids research* Vol. 40 4288-4297 (2012).
5 Subramanian, A. *et al.* Gene set enrichment analysis: a knowledge-based approach for interpreting genome-wide expression profiles. *Proceedings of the National Academy of Sciences of the United States of America* **102**, 15545-15550, doi:10.1073/pnas.0506580102 (2005).
6 Han, J. *et al.* in *Sci Rep* Vol. 7 (2017).
7 Hellemans, J., Mortier, G., De Paepe, A., Speleman, F. & Vandesompele, J. qBase relative quantification framework and software for management and automated analysis of real-time quantitative PCR data. *Genome Biol* **8**, R19, doi:10.1186/gb-2007-8-2-r19 (2007).

Supplementary tables

Table S1. **Patient characteristics**. None of the patients carried a germline *PTPN11* or *CBL* mutation and patients were treated with standard of care therapy.

Table S2. ***In house* designed primers and conditions used for qPCR assays on selected lncRNAs.**

Table S3. **Differentially expressed lncRNAs in JMML vs PNBM using EdgeR**.

Table S4. **Differentially expressed lncRNAs in molecular subgroups vs PNBM using EdgeR.**

Table S5. **Hematopoietic cell lines with expression of lncRNA based on CCLE RNA sequencing data and validated by qPCR.**

Table S1. Patient characteristics: None of the patients carried a germline PTPN11 or *CBL* mutation and patients were treated with standard of care therapy.

abbreviations: BM = bone marrow; PB = peripheral blood; Del = chromosomal deletion; TRM = transfusion related mortality, HSCT = haematopoietic stem cell transplantation; GVH = graft versus host

ID	Sample	Sex	Age at diagnosis (months)	HbF %	Age-adjusted HbF	Karyotype	Mutation	BM blast %	Platelets (x 10^9/L)	Cause of death	Survival from diagnosis (OS) (years)	Events after HSCT	Survival from HSCT (EFS) (years)	included in cohort
G2	B013	F	15	2.2	Elevated	46,XX	KRAS	6.0	70		5.58	No event	5.19	Discovery
G7	B034	M	36	47.00	Elevated	46,XY	KRAS	7.2	21		2.28	No event	2.09	Discovery
C6	CZ076	M	25	37.0	Elevated	46,XY	KRAS	0.0	25		4.12	No event	3.98	Discovery
C8	CZ022	M	43	17.1	Elevated	46,XY	NF1	8	179	TRM	1.28	TRM	0.20	Discovery
D2	D102	M	45	59.5	Elevated	46,XY	NF1	13.0	103	Progress, no SCT	0.79	no SCT	no SCT	Discovery
F10	F10	F	159	0.2	Normal	45,XX,-7	NRAS	18	180		2.03	No event	1.89	Discovery
F11	F11	M	50	/	No data	46,XY	NRAS	17.0	63		4.20	No event	3.81	Discovery
F12	F12	F	8	0.3	Normal	45,XX,-7[30] / 46,XX[1]	KRAS	0	58		5.38	No event	4.77	Discovery
F15	F15	F	34	31.8	Elevated	Del(6)	NRAS	37.0	72		3.13	No event	2.91	Discovery
F16	F16	F	54	58.5	Elevated	Del(7)	NRAS	15	197	Disease progression and respiratory failure	1.57	Relapse	0.18	Discovery
F17	F17	M	62	0.4	Elevated	46,XY	NRAS	3.0	84		6.50	no SCT	no SCT	Discovery
F25	F25	M	1	31.9	Normal	46,XY	PTPN11	6	80		2.93	No event	2.16	Discovery
F26	F26	M	3	36.0	Elevated	46,XY	PTPN11	0.0	33		0.79	No event	0.36	Discovery
F27	F27	M	23	26.9	Elevated	46,XY	PTPN11	4.5	50		1.68	No event	1.29	Discovery
F4	F4	M	26	/	No data	46,XY,del9(q21q32)[10] / 48,idem,+21,+22 [1] / 48,XY,+21,+22 [3] / 46,XY[16]	PTPN11	32.0	52	Disease progression (acutisation, relapse after second HSCT)	2.06	Relapse	0.62	Discovery
F5	F5	F	8	7.4	Elevated	46,XX	PTPN11	7	79		4.33	No event	3.79	Discovery
F9	F9	M	23	37.0	Elevated	46,XY	PTPN11	3.5	25		1.69	No event	1.32	Discovery
NL10	NL043	M	39	35	Elevated	46,XY	PTPN11	2	66		15.10	Relapse	0.82	Discovery
NL11	NL072	M	10	1.2	Normal	45,XY,-7	PTPN11	12.0	23	Relapse	1.27	Relapse	0.30	Discovery
G4	B009	M	19	50.00	Elevated	complex	PTPN11	9.5	8	Relapse	0.62	Relapse	0.11	Validation
G6	B014	M	49	36.1	Elevated	Normal	PTPN11	3.0	29		5.74	No event	5.43	Validation
NL5	NL109	F	17	4.30	Elevated	45,XX,-7	Quad neg	18.0	204		7.21	No event	6.51	Validation
NL6	NL118	M	6	1.0	Normal	45,XY,-7	KRAS	1.0	30	Progress, no SCT	0.06	no SCT	no SCT	Validation
NL7	NL105	M	2	43.8	Normal	Normal	KRAS	2	31		6.32	No event	6.01	Validation
D3	D127	M	65	5.9	Elevated	46,XY	NF1	0.0	139	Relapse	1.82	Relapse	0.49	Validation
D8	D311	M	35	51.9	Elevated	46,XY	quintuple negative	5	146	Relapse (TRM after 2nd SCT)	2.27	Relapse	1.44	Validation
F13	F13	M	40	69.5	Elevated	No data	NRAS	19.0	34	HSCT complication	0.60	TRM	0.24	Validation
F34	F34	M	19	/	No data	46,XY	NRAS	3	217		3.12	No event	2.37	Validation
F3	F3	M	26	37.4	Elevated	Del(Y)	PTPN11	11.0	46	Toxicity (severe veino occlusive disease after HSCT)	0.38	TRM	0.09	Validation
F1	F1	F	54	5.6	Elevated	47,XX,+21	PTPN11	7	98	Articular GVH, death due to septic shock after surgery	4.84	TRM	4.22	Validation
D1	D360	M	64	/	No data	45,XY,-21	KRAS	1.0	21	Progress, no SCT	0.50	no SCT	no SCT	Validation + primary culture
D10	D120						PTPN11							primary culture
G9	FG	M	10	3.7	Elevated	45,XY,-7	PTPN11	25.0	84		1.20	no SCT	no SCT	primary culture

Table S2. In house designed primers and conditions used for qPCR assays on selected lncRNAs

abbreviations: qPCR = quantitative reverse transcriptase PCR; lncRNA = long non-coding RNA

lncRNA_transcript	lncRNA_gene	ensembl_gene_id	gene	Primer forward	Primer reverse	qPCR efficiency (%)	Selection
lnc-THADA-4:3	lnc-THADA-4	ENSG00000224739.2	AC016735.1	CCCAGAGACGTGAAGCCA	GAGGCCCCTGTTTCTCACC	94	RNAseq + microarray
lnc-THADA-4:1	lnc-THADA-4	ENSG00000224739.2	AC016735.1	TCTGGGCTCACTACATGGGA	TCAGGTCCTCTCCCTCCTTG	95	RNAseq + microarray
lnc-RUNX3-4:1	lnc-SYF2-2	ENSG00000233755.1	RP4-799D16.1	GCCTCAACCAGCGTTCTCTA	TCCTCTGTCAGATGCCAACAC	96	microarray
lnc-BASP1-3:1 = LINC02217:30	lnc-BASP1-3 = LINC02217	ENSG00000248455.1	/	GCAAGCATCAGTACAGAAGTCG	GCATCAAAGGGTCTACTGGAAT	95	microarray
lnc-BASP1-4:5 = LINC02218:5	lnc-BASP1-4 = LINC02218	ENSG00000249662.5	RP11-321E2.4	GTTCCAGACTGTTTCCAAGATTG	TCTCTTGCCTTCCAACTGCA	89	RNAseq
lnc-ACSL1-1:1	lnc-ACSL1-1	ENSG00000251230.1	MIR3945HG	AGGTCACTTGTCTAGCCCCT	TGCTGCCTTGAATTGGGACA	93	RNAseq + microarray
lnc-ACSL1-1:4	lnc-ACSL1-1	ENSG00000251230.5	MIR3945HG	CCAGTGCCAGGAAAACAGGA	TGAGAGCATTGCCACCATGT	95	RNAseq + microarray
lnc-CROCC-1:4	lnc-CROCC-1	ENSG00000245549	AL137798.2	GTGGGAAACGGTGTTTGTGC	TGAGGCGTTGTCAGTCTTGA	98	RNAseq
lnc-KCNA5-2:1	lnc-KCNA5-2	ENSG00000256790	/	CCACCCTCCTCCTTGTCTTC	GTGACCATGACCTCCCAGAG	94	RNAseq
NRIR:1	NRIR	ENSG00000225964.5	NRIR	GTGACATGGTTTTCTGGTGCC	AGAGGTGTCTGCTGCAATAAT	95	RNAseq subgroup analysis
NRIR:2	NRIR	ENSG00000225964.5	NRIR	CATGGTTTTCTGGTGCCTTGG	CAGATTAAGGAGGTTAGAGGTGT	92	RNAseq
lnc-MDGA2-2:1	lnc-MDGA2-2	/	/	AGTCACAGAAGGAACAGACACT	TCCTCATCTTCCCCTCTGCT	96	RNAseq
lnc-POC1A-1:1	lnc-POC1A-1	/	/	TGAGTGACAAGGACAGTGGC	CCACCAGGTCTATGCCCTAA	92	differential isoform ballgown
lnc-THNSL1-2:1	lnc-THNSL1-2	ENSG00000238115.1	PRINS	GCAGACAGGAGCAAGGTACA	CACAGCAGTTTAGGGACCCC	92	RNAseq
MEG3:1	MEG3	ENSG00000214548.14	MEG3	AAGGACCACCTCCTCTCCAT	AGGAAACCGTGCTCCTAGTG	94	RNAseq
MEG8:1	MEG8	ENSG00000232018.4	MEG8	TGTCGGAGGATCGTGTCAT	AATCTTCTAGAGCCCCAGATCC	94	RNAseq
lnc-EGR3-1:1	lnc-EGR3-1	ENSG00000261026.1	CTD-3247F14.2	TTCCACAGTTTCAAGCATGG	CCACAGCCTTGGTCTTTTGT	94	microarray
lnc-ACOT9-1:1	lnc-ACOT9-1	ENSG00000233785.1	RP13-314C10.5	CCAAAGGAATGTGAGCTCGT	GGGCGGTGGTAATAGTGACA	93	microarray
MKLN1-AS:1	MKLN1-AS	ENSG00000236753.5	MKLN1-AS	GACTCCTGAAAGCCTGGACA	TCACTGTGAATCCTGCTGACTT	95	RNAseq
MIR222HG:12 = lnc-CXorf36-46:1	MIR222HG = lnc-CXorf36-46	ENSG00000231566.1	RP5-1158E12.3	CTTTCTTGGAGTCCGCACAC	GATCACACGAGGCGCTTTTC	95	RNAseq
TMEM202-AS1:1	TMEM202-AS1	ENSG00000261187.1	RP11-1007O24.2	GAGTTCTTGGTCATGGTGCC	CGGGACTTGCTATGTTGCTC	94	microarray
lnc-CLEC4E-1:1	lnc-CLEC4E-1	ENSG00000255801.1	RP11-561P12.5	GGGCTCATCTGGGATCTCC	AGTGAAGAGGGACAACATGGT	92	microarray
SNHG15:10	SNHG15	ENSG00000232956.3	SNHG15	AGTCTCATGTTCAAGCAGTCTT	AGAAACACTGACGGATGGCA	87	microarray
lnc-CYP4F22-4:1	lnc-CYP4F22-4	ENSG00000270802.2	/	GGAATGCCCCACAACTGTTA	TGCCCTTAACTTGTCCGTTT	96	microarray
lnc-FCAR-1:1	lnc-FCAR-1	ENSG00000268734.1	CTB-61M7.2	TGTTGAAGTGAGGCTCTCCC	GGCTGAGGTAGGAGGATTGC	88	RNAseq subgroup analysis (KRAS, monosomy7)
lnc-OSR2-2:1	lnc-OSR2-2	ENSG00000253532.1	CTD-2340D6.1	TTTAAGTCACCGATCCTGGC	ACATGATGAAACCCTGTCCCT	96	RNAseq subgroup analysis
lnc-PARVA-7:1	lnc-PARVA-7	ENSG00000254983.1	RP11-573E11.2	ACTTTGAGAGGCTAGTCTTGAAC	GCTCACACCTATAATCCCAGCA	88	RNAseq
lnc-BCAR1-1:1	lnc-BCAR1-1	ENSG00000259999.1	RP11-252K23.1	GCTGGAGTGCAATGGTGTGA	TCCACTTCCCAGGTTCATGC	89	RNAseq
lnc-TOMM20-1:1	lnc-TOMM20-1	ENSG00000258082.1	RP11-443B7.3	GGACCCTATGACCTTGCTTCA	GTGTCAGCAGCGATGTCT	95	RNAseq
lnc-TOMM20-1:2	lnc-TOMM20-1	ENSG00000258082.1	RP11-443B7.3	GGTAGGAGGAAGTGGATGCG	AGAAGAGGTTTTAGGTGTCAGCA	94	differential isoform ballgown
lnc-ADH5-1:1	lnc-ADH5-1	ENSG00000272777.1	RP11-571L19.8	TTGACTCAGGGAAGGGGAAAC	GCAGATTGGTAGCCGAGCA	97	RNAseq
lnc-ANTXR2-1:2	lnc-ANTXR2-1	ENSG00000248719.1	RP11-377G16.2	CAGCCTGCAAGTTGACTCCT	GTGGGGCTGAAGGAGTTGAG	91	RNAseq
lnc-LTF-1:1	lnc-LTF-1	ENSG00000223552.1	RP11-24F11.2	CCACCCGCTGATTCAATACG	ATCATGTAGGTGCAGGCAGA	93	RNAseq
lnc-COL4A1-5:1	lnc-COL4A1-5	/	MSTRG.45275.7	ACATGCTTGTTGGGGAAGAG	AGCTGATCACCAGTCCTTGC	89	RNAseq
lnc-LIPC-3:1	lnc-LIPC-3	/	NONHSAT044099	ACACATCATACACCAACGCC	CAGTTGTGAAGCGAGGTTGT	94	RNAseq
lnc-LIPC-4:1	lnc-LIPC-4	/	NONHSAT044098	GAAGCTTTACAGGTGCAGTCA	TGGACTCGCTCGTAGTTTGA	93	RNAseq
lnc-LIPC-6:1	lnc-LIPC-6	/	NONHSAT044096	AGGCATATACTTCACCCTCCT	AAGTCCATGAAAGCCTGTGG	92	RNAseq
lnc-MARCH7-1:1	lnc-MARCH7-1	ENSG00000224152.1	AC009506.1	GAAACCCCACCACCTCCTTA	CAGAGAGAGAGAGGCGGAAG	91	differential isoform ballgown
lnc-EDAR-2:1 = SH3RF3-AS1:2	SH3RF3-AS1	ENSG00000259863.1	SH3RF3-AS1	AGGATCTCGTTCTGTCAGGC	TCAGGAGGCTGAGGTGAAAG	95	differential isoform ballgown
GASAL1:1	GASAL1	ENSG00000253669.3	KB-1732A1.1	CTGAGGCCAAAGTTTCCAAC	CAGCCTGACTTTCCCTCTTCT	93	differential isoform ballgown
TPT1-AS1:1	TPT1-AS1	ENSG00000170919.9	TPT1-AS1	CACTCCCAGATCTTCACTTCAGG	AATTGGAGGCCAGTGCTCTG	94	differential isoform ballgown
lnc-MAP7D2-1:1	lnc-MAP7D2-1	ENSG00000246650	AL732366.1	TGGGCATGGTGGTTCATACT	GTCTTGCTATGTTGCCAGGG	93	microarray
LINC01270:1	LINC01270	ENSG00000203999.4	RP11-290F20.1	TGTGGCTGATCTGCTTTCTG	GAAGCATCTGGGCTAACTGG	91	microarray
LINC02506:1	LINC02506	ENSG00000251129.1	RP11-734I18.1	CACACTACCAAAGGCAGAGG	GCGACCATTTCCTGACACTT	94	microarray
lnc-ATAD5-2:1	lnc-ATAD5-2	ENSG00000263603.1	CTD-2349P21.5	CAGGGAAGGTGAGGAATCAA	GCTGGGATTACTGCTTGAGG	94	RNAseq
lnc-GOLGA8K-1:1	lnc-GOLGA8K-1	ENSG00000215304.2	RP13-395E19.3	ACTGCGATATATGGGCCAAG	ATGAGGGCTTTAGGTCAGCA	92	RNAseq
lnc-SPOPL-18:1	lnc-SPOPL-18	ENSG00000237772.1	AC092620.1	GGCTGTTGATCCACTGAACC	TTGCCAAGATGTAGCCCATA	98	RNAseq
lnc-TAOK1-5:1	lnc-TAOK1-5	ENSG00000266111.1	RP11-296K13.4	CCCACCTCAGCCTCCTAAGT	TGCAGTGAGCTGTGATTGTG	88	RNAseq
XIST:1	XIST	ENSG00000229807.10	LINC00001	GGATGTCAAAAGATCGGCCC	GTCCTCAGGTCTCACATGCT	93	RNAseq

Name	Type	Primer forward	Primer reverse
MALAT1	Efficient LNA GapmeR knockdown	GGATTCCAGGAAGGAGCGA G	ATTGCCGACCTCAC GGATTT
GAPD	primers housekeeping gene	GGCATGGACTGTGGTCATGA G	GGCATGGACTGTGG TCATGAG
HPRT1	primers housekeeping gene	TGACACTGGCAAAACAATG CA	GGTCCTTTTCACCA GCAAGCT
TBP	primers housekeeping gene	CGGCTGTTTAACTTCGCTTC	CACACGCCAAGAA ACAGTGA

Table S3. differentially expressed lncRNAs in JMML vs PNBM using EdgeR

abbreviations: lncRNA = long non-coding RNA; JMML = juvenile myelomonocytic leukemia; PNBM = pediatric normal bone marrow; FC = fold change; adj.P = adjusted P-value

Ensembl_gene	LNCipedia_name	logFC	AveExpr	t	B	adj.P_EdgeR	chx	start	stop	dir	gene_type	gene
ENSG00000255801.1	lnc-CLEC4E-1	5.76	3.38	5.87	5.45	0.000599	chr12	8548361	8567613	-	lincRNA	RP11-561P12.5
ENSG00000261026.1	lnc-EGR3-1	5.69	6.24	3.25	-1.55	0.044105	chr8	22679013	22684009	-	sense_overlapping	CTD-3247F14.2
ENSG00000263050.1	lnc-SMG6-2	5.50	3.69	6.24	6.39	0.000373	chr17	2043475	2044968	-	lincRNA	RP11-667K14.3
ENSG00000233785.1	lnc-ACOT9-1	5.19	2.89	8.65	12.91	0.000005	chrX	23772992	23782956	-	antisense	RP13-314C10.5
ENSG00000236753.5	MKLN1-AS	4.92	3.85	5.49	3.96	0.001310	chr7	131309744	131328222	-	processed_transcript	MKLN1-AS
ENSG00000223662.1	/	4.81	2.66	6.00	5.80	0.000476	chr21	14582202	14598303	+	antisense	SAMSN1-AS1
ENSG00000226644.5	lnc-STK35-4	4.76	3.01	4.91	2.71	0.004119	chr20	2207217	2213151	+	lincRNA	RP11-128M1.1
ENSG00000230257.1	/	4.72	2.56	5.08	3.23	0.002933	chr7	102973522	102988856	+	antisense	NFE4
ENSG00000253532.1	lnc-OSR2-2	4.59	3.07	5.75	4.89	0.000739	chr8	99119352	99120581	+	sense_intronic	CTD-2340D6.1
ENSG00000243179.1	LINC01191	4.59	2.42	5.07	3.18	0.003043	chr2	113979909	113983258	+	lincRNA	AC110769.3
ENSG00000233061.1	TTLL7-IT1	4.47	2.24	5.39	4.08	0.001662	chr1	83979118	83984300	-	sense_intronic	TTLL7-IT1
ENSG00000203999.8	LINC01270	4.47	3.74	3.60	-0.75	0.045866	chr20	50292720	50314922	+	lincRNA	LINC01270
ENSG00000254983.1	lnc-PARVA-7	4.42	2.26	6.17	6.26	0.000374	chr11	12303533	12308216	+	sense_intronic	RP11-573E11.2
ENSG00000249662.5	LINC02218	4.41	2.28	5.50	4.40	0.001310	chr5	17444010	17483946	+	lincRNA	RP11-321E2.4
ENSG00000263603.1	lnc-ATAD5-2	4.38	2.17	6.25	6.48	0.000373	chr17	30729469	30731202	+	lincRNA	CTD-2349P21.5
ENSG00000237772.1	lnc-SPOPL-18	4.34	5.68	3.29	-1.47	0.040921	chr2	138604510	138613167	+	lincRNA	AC092620.3
ENSG00000249464.5	lnc-SPRY1-11	4.28	2.13	5.21	3.59	0.002392	chr4	123650267	123930406	+	lincRNA	LINC01091
ENSG00000259999.1	lnc-BCAR1-1	4.25	2.07	6.47	7.10	0.000266	chr16	75321890	75325048	-	sense_intronic	RP11-252K23.1
ENSG00000249309.1	lnc-TLR2-1	4.20	3.77	3.59	-0.77	0.046272	chr4	153720327	153727722	+	antisense	RP11-153M7.5
ENSG00000253619.1	lnc-MTBP-2	4.08	1.96	5.90	5.50	0.000562	chr8	120913065	121119754	+	lincRNA	RP11-369K17.1
ENSG00000224739.2	lnc-THADA-4	4.02	5.04	2.83	-2.44	0.001280	chr2	43001355	43006060	-	lincRNA	AC016735.1
ENSG00000237372.2	lnc-GADD45G-3	3.98	1.85	4.67	2.09	0.006709	chr9	89639814	89719759	+	lincRNA	UNQ6494
ENSG00000272269.1	/	3.97	3.40	3.76	-0.37	0.036403	chr6	17706257	17707344	+	antisense	RP11-500C11.3
ENSG00000234199.2	LINC01191	3.95	1.81	4.92	2.77	0.004119	chr2	113979569	114007302	+	lincRNA	LINC01191
ENSG00000258499.1	LINC02287	3.92	1.80	5.70	4.93	0.000844	chr14	92905697	92907482	-	lincRNA	RP11-862G15.2
ENSG00000231842.1	lnc-TRMT11-2	3.91	1.77	5.98	5.72	0.000488	chr6	126177193	126202239	+	lincRNA	RP11-527F13.1
ENSG00000271133.5	lnc-MACC1-3	3.90	1.77	5.09	3.23	0.002933	chr7	20328299	20331747	-	antisense	CTA-293F17.1
ENSG00000235770.5	LINC00607	3.90	1.78	5.14	3.39	0.002738	chr2	215611563	215843722	-	lincRNA	LINC00607
ENSG00000233554.5	/	3.90	1.75	5.62	4.71	0.000998	chr9	33166975	33179983	+	antisense	B4GALT1-AS1
ENSG00000236914.3	LINC01852	3.89	1.80	5.17	3.46	0.002554	chr15	38069481	38072959	-	lincRNA	RP11-1008C21.2
ENSG00000215304.3	lnc-GOLGA8K-1	3.88	2.80	4.00	0.29	0.024111	chr15	32398956	32435233	-	processed_transcript	RP13-395E19.3
ENSG00000228315.11	lnc-IGLL1-1	3.74	1.63	6.03	5.86	0.000472	chr22	23638487	23717356	-	processed_transcript	GUSBP11
ENSG00000274092.1	lnc-NSMCE1-11	3.65	1.58	4.77	2.37	0.005349	chr16	27313387	27314101	-	antisense	CTD-3203P2.3
ENSG00000278727.1	lnc-ACOD1-5	3.64	1.55	5.38	4.05	0.001662	chr13	76887551	76891135	+	lincRNA	AC000403.4
ENSG00000228022.5	HCG20	3.64	1.57	4.39	1.33	0.010695	chr6	30766825	30792250	+	lincRNA	HCG20
ENSG00000257550.1	/	3.57	1.50	4.58	1.83	0.007910	chr12	53513984	53517608	+	antisense	RP11-793H13.3
ENSG00000203801.8	LINC00222	3.56	1.51	4.49	1.60	0.008888	chr6	108751654	108769942	+	lincRNA	LINC00222
ENSG00000261187.1	TMEM202-AS1	3.55	1.49	5.25	3.69	0.002220	chr15	72465128	72466262	-	lincRNA	RP11-1007O24.2
ENSG00000256571.1	lnc-RXYLT1-1	3.54	1.47	4.98	2.95	0.003622	chr12	63878787	63879474	+	sense_intronic	RP11-274J7.2
ENSG00000259269.1	lnc-THBS1-1	3.54	1.46	4.31	1.11	0.012576	chr15	39472703	39481206	+	lincRNA	RP11-624L4.2
ENSG00000271806.1	lnc-FAAP20-3	3.54	2.20	3.78	-0.26	0.035607	chr1	2141084	2145279	-	antisense	RP5-892K4.1
ENSG00000225329.3	LHFPL3-AS2	3.53	1.44	5.42	4.15	0.001596	chr7	104894628	104926645	-	processed_transcript	LHFPL3-AS2
ENSG00000249173.5	LINC01093	3.52	1.47	4.23	0.89	0.014507	chr4	184893871	184899454	-	lincRNA	LINC01093
ENSG00000230753.5	ZNF341-AS1	3.51	1.46	4.45	1.49	0.009738	chr20	33787373	33811097	-	antisense	ZNF341-AS1
ENSG00000261916.1	lnc-SHPK-1	3.50	1.42	4.66	2.05	0.006709	chr17	3601761	3602272	-	sense_intronic	RP11-235E17.4
ENSG00000248719.1	lnc-ANTXR2-1	3.50	2.34	4.28	1.02	0.013618	chr4	80183280	80190169	-	antisense	RP11-377G16.2
ENSG00000260070.1	lnc-EEPD1-1	3.45	1.39	4.53	1.70	0.008478	chr7	36319775	36320479	+	antisense	RP11-182J23.1
ENSG00000198685.3	LINC01565	3.44	1.37	4.84	2.56	0.004644	chr3	128572000	128576086	-	lincRNA	LINC01565
ENSG00000203364.2	lnc-CARD19-4	3.43	1.36	4.23	0.89	0.014507	chr9	93147040	93148556	+	lincRNA	RP11-370F5.4
ENSG00000227938.1	lnc-TRMT61B-3	3.43	1.38	4.12	0.60	0.018513	chr2	28448167	28450184	-	lincRNA	AC104695.4
ENSG00000271959.1	/	3.39	1.33	5.21	3.59	0.002392	chr8	141434545	141437954	+	antisense	CTD-3064M3.7
ENSG00000259473.1	LINC02205	3.39	1.33	4.80	2.43	0.005117	chr15	70503907	70505928	-	lincRNA	RP11-96C21.1
ENSG00000253837.1	/	3.38	1.32	4.63	1.97	0.007161	chr8	23336171	23366125	+	processed_transcript	RP11-177H13.2
ENSG00000270190.1	lnc-ANAPC1-5	3.38	1.32	4.92	2.76	0.004119	chr2	111266868	111267473	-	lincRNA	RP11-803D5.4
ENSG00000248884.1	lnc-CCDC125-6	3.37	1.33	4.28	1.02	0.013603	chr5	68430427	68434481	-	lincRNA	CTC-537E7.3
ENSG00000227911.3	LINC02344	3.34	1.74	4.00	0.29	0.024266	chr13	33333679	33335277	-	lincRNA	RP11-141M1.1
ENSG00000230490.2	lnc-STARD13-2	3.34	1.28	3.98	0.23	0.025023	chr13	33355206	33676768	-	lincRNA	RP11-141M1.3
ENSG00000257696.1	lnc-ACTR6-1	3.33	1.29	4.47	1.54	0.009389	chr12	100143058	100144671	+	antisense	RP11-175P13.2
ENSG00000261602.1	lnc-NFAT5-1	3.31	1.26	4.84	2.55	0.004664	chr16	69709874	69710583	+	antisense	CTD-2033A16.1
ENSG00000264812.1	lnc-C17orf62-3	3.31	1.74	4.24	0.92	0.014333	chr17	82400703	82401382	-	sense_intronic	RP13-20L14.4
ENSG00000225822.4	/	3.31	1.26	5.02	3.06	0.003328	chr3	196431385	196432530	+	antisense	UBXN7-AS1
ENSG00000280474.1	lnc-GLE1-6	3.29	1.24	4.57	1.81	0.007952	chr9	128595893	128596633	+	sense_intronic	RP11-216B9.8
ENSG00000224652.1	LINC00885	3.28	1.24	4.44	1.45	0.010025	chr3	196142636	196160890	+	lincRNA	LINC00885
ENSG00000253111.1	lnc-TRIB1-2	3.26	1.21	4.16	0.69	0.017004	chr8	125466939	125541373	+	lincRNA	RP11-136O12.2
ENSG00000264695.1	lnc-HRH4-1	3.23	1.20	3.95	0.15	0.026526	chr18	24489375	24491080	+	lincRNA	RP11-178F10.2
ENSG00000257156.1	LINC02458	3.22	1.18	3.59	-0.79	0.046272	chr12	89048187	89309553	-	lincRNA	RP11-13A1.3
ENSG00000251413.1	lnc-LRRC18-3	3.22	1.20	4.18	0.75	0.016368	chr10	48624004	48625093	-	sense_intronic	RP11-534L6.5
ENSG00000224187.1	LINC01991	3.20	1.17	4.21	0.84	0.014993	chr3	187958775	187976407	-	lincRNA	RP11-132N15.3
ENSG00000261573.1	lnc-ATP6V1G3-2	3.19	1.16	4.14	0.66	0.017508	chr1	198657553	198667061	-	antisense	RP11-553K8.5
ENSG00000268628.2	lnc-CRNKL1-8	3.18	1.15	4.58	1.84	0.007910	chr20	19756390	19758037	-	lincRNA	RP1-122P22.4
ENSG00000205786.8	LINC01531	3.17	1.13	3.85	-0.10	0.031571	chr19	35399511	35419385	+	lincRNA	LINC01531
ENSG00000228221.5	LINC00578	3.16	1.14	4.37	1.27	0.011152	chr3	177441921	177752305	+	lincRNA	LINC00578
ENSG00000274363.1	lnc-CYB5D2-4	3.12	1.10	4.34	1.17	0.011945	chr17	4267691	4268430	+	lincRNA	RP11-104O19.4
ENSG00000272620.1	/	3.11	1.08	4.34	1.18	0.011945	chr4	7754090	7778928	+	antisense	AFAP1-AS1
ENSG00000253315.1	LINC01932	3.05	1.03	4.00	0.28	0.024111	chr5	159227715	159245127	+	lincRNA	CTB-11I22.2
ENSG00000259418.1	lnc-DUOX2-2	3.01	0.99	3.68	-0.56	0.040584	chr15	45041716	45058707	-	antisense	RP11-109D20.1
ENSG00000251443.1	LINC02160	3.00	1.01	3.61	-0.74	0.045094	chr5	33229735	33255553	+	lincRNA	RP11-113I22.1
ENSG00000229162.1	lnc-CLIC4-1	2.98	1.46	3.54	-0.88	0.049581	chr1	24961345	24963097	+	antisense	RP11-84D1.1
ENSG00000268170.2	lnc-FAM131B-2	2.97	0.97	3.89	-0.02	0.030177	chr7	143220468	143222267	-	antisense	RP11-556I13.2
ENSG00000226576.1	lnc-WDFY4-1	2.97	0.96	4.21	0.84	0.014993	chr10	48984564	49018897	+	antisense	RP11-523O18.1
ENSG00000260988.1	lnc-SH2D7-1	2.96	0.96	3.80	-0.23	0.034540	chr15	78141243	78143173	+	lincRNA	RP11-285A1.1
ENSG00000259422.1	lnc-ETFA-1	2.95	0.96	4.04	0.39	0.021998	chr15	76174891	76181486	-	antisense	RP11-593F23.1
ENSG00000237845.1	lnc-NID1-2	2.95	0.94	3.87	-0.07	0.031279	chr1	235942553	235943805	-	lincRNA	RP5-940F7.2
ENSG00000224276.1	lnc-RXFP4-1	2.94	0.93	3.92	0.06	0.028262	chr1	155991390	156001787	+	antisense	RP11-336K24.5
ENSG00000258760.1	lnc-MAX-2	2.92	0.93	3.86	-0.09	0.031571	chr14	65236480	65318790	-	lincRNA	CTD-2509G16.5
ENSG00000259293.1	LIPC-AS1	2.91	0.88	4.03	0.36	0.022456	chr15	58434890	58498735	-	antisense	LIPC-AS1
ENSG00000258976.1	lnc-LTBP2-1	2.89	0.88	3.92	0.05	0.028262	chr14	74614693	74616647	-	lincRNA	CTD-2207P18.2
ENSG00000236519.1	LINC01424	2.88	0.90	4.26	0.96	0.014015	chr21	44802577	44804717	+	antisense	LINC01424
ENSG00000233431.1	LINC02596	2.87	0.88	3.70	-0.50	0.039508	chr1	21586472	21591187	+	antisense	RP11-63N8.3
ENSG00000265010.1	lnc-COG1-1	2.85	0.87	3.96	0.16	0.026239	chr17	73243093	73244706	+	sense_intronic	RP11-661C3.2
ENSG00000224969.1	lnc-HES4-1	2.83	0.86	3.65	-0.63	0.042314	chr1	1011997	1013193	-	antisense	RP11-54O7.11
ENSG00000278095.1	lnc-ITPR2-5	2.83	0.84	3.89	-0.02	0.030177	chr12	26273329	26273900	-	antisense	RP11-283G6.6
ENSG00000261071.1	lnc-RANBP9-1	2.83	1.30	3.54	-0.88	0.049597	chr6	13614111	13615155	-	antisense	RP1-223E5.4

ENSG00000254739.1	lnc-LRRC56-1	2.81	0.84	4.33	1.17	0.011945	chr11	528907	529659	+	antisense	RP13-46H24.1
ENSG00000271797.1	/	2.81	1.33	3.58	-0.77	0.046524	chr5	115262505	115263448	+	antisense	CTC-428G20.6
ENSG00000280924.1	LINC00628	2.78	0.78	3.54	-0.92	0.049581	chr1	204368431	204369719	-	lincRNA	LINC00628
ENSG00000226004.1	lnc-PERP-1	2.78	0.79	3.66	-0.60	0.041279	chr6	137943079	137945802	-	lincRNA	RP11-10J5.1
ENSG00000274029.1	lnc-P2RX7-7	2.78	0.81	3.72	-0.46	0.039067	chr12	121190868	121191518	+	lincRNA	RP11-340F14.6
ENSG00000236495.1	lnc-HSPA14-1	2.77	0.78	3.80	-0.24	0.034602	chr10	14652742	14661691	+	antisense	RP11-7C6.1
ENSG00000231729.1	ARHGEF9-IT1	2.74	0.79	3.75	-0.37	0.037090	chrX	63670196	63671502	-	sense_intronic	ARHGEF9-IT1
ENSG00000249550.6	LINC01234	2.73	0.74	3.73	-0.43	0.038158	chr12	113679459	113773683	-	lincRNA	LINC01234
ENSG00000275383.1	lnc-ZFP90-4	2.73	0.77	4.17	0.72	0.016594	chr16	68591382	68594424	+	lincRNA	RP11-615I2.6
ENSG00000255314.2	lnc-CREB3L1-2	2.73	0.72	3.78	-0.30	0.035627	chr11	46123573	46177106	+	lincRNA	RP11-702F3.3
ENSG00000235865.2	GSN-AS1	2.70	0.72	3.79	-0.28	0.035489	chr9	121280768	121285530	-	antisense	GSN-AS1
ENSG00000253139.1	lnc-PENK-1	2.70	0.73	3.94	0.13	0.026546	chr8	56536758	56540497	-	lincRNA	RP11-17A4.3
ENSG00000269145.2	lnc-RAB3A-3	2.70	0.69	3.70	-0.50	0.039508	chr19	18144522	18151691	-	antisense	AC007192.6
ENSG00000272555.1	lnc-CYP27A1-1	2.69	0.71	3.61	-0.73	0.044999	chr2	218818690	218819144	+	lincRNA	RP11-459I19.1
ENSG00000267784.1	/	2.68	0.70	3.67	-0.59	0.041157	chr2	178723457	178727046	+	antisense	RP11-171I2.1
ENSG00000232324.1	lnc-MYADM-1	2.67	0.66	3.94	0.12	0.026803	chr19	53864763	53866140	+	lincRNA	AC008440.10
ENSG00000204528.3	PSORS1C3	2.66	0.69	3.69	-0.53	0.039782	chr6	31173735	31177899	-	sense_intronic	PSORS1C3
ENSG00000266903.1	lnc-CEACAM20-8	2.66	0.72	3.70	-0.51	0.039592	chr19	44632199	44718759	-	antisense	CTB-171A8.1
ENSG00000259182.5	lnc-LRRK1-2	2.66	0.68	3.74	-0.40	0.037689	chr15	101168530	101170821	+	lincRNA	RP11-424I19.2
ENSG00000257953.1	lnc-TSFM-1	2.66	0.71	3.81	-0.23	0.034540	chr12	57837092	57842745	+	antisense	RP11-620J15.1
ENSG00000203279.3	lnc-CTSV-5	2.63	0.68	3.76	-0.36	0.036630	chr9	97200475	97238700	-	lincRNA	RP11-498P14.5
ENSG00000267882.2	lnc-EYA2-3	2.62	0.63	3.76	-0.35	0.036630	chr20	47318502	47320754	+	antisense	RP4-569M23.5
ENSG00000254981.1	lnc-FGFR1-1	2.61	0.64	3.65	-0.63	0.042244	chr8	38400536	38401683	-	antisense	RP11-350N15.3
ENSG00000260896.5	ARLNC1	2.61	0.68	3.67	-0.58	0.041025	chr16	80828735	80892595	-	lincRNA	PRCAT47
ENSG00000226676.1	NUTM2B-AS1	2.61	0.67	3.59	-0.79	0.046238	chr10	79628757	79632188	-	lincRNA	RP11-589B3.6
ENSG00000223795.2	lnc-RXRA-3	2.59	0.60	3.60	-0.75	0.045213	chr9	134505472	134521442	+	lincRNA	RP11-473E2.2
ENSG00000225964.5	NRIR	2.58	6.18	1.97	-4.15	0.026200	chr2	6828514	6840464	-	antisense	NRIR
ENSG00000225235.1	INTS6L-AS1	2.56	0.61	3.61	-0.75	0.045213	chrX	135520083	135520674	-	antisense	INTS6L-AS1
ENSG00000277159.1	lnc-TUBGCP3-20	2.55	0.62	3.68	-0.56	0.040531	chr13	112602828	112606417	-	lincRNA	RP11-88E10.4
ENSG00000258354.1	lnc-NTAN1-2	2.51	0.58	3.55	-0.88	0.048640	chr16	14909887	14911345	-	lincRNA	MIR3180-1
ENSG00000259416.2	lnc-KLHL25-1	2.49	0.54	3.58	-0.81	0.046524	chr15	85754941	85756237	-	antisense	RP11-158M2.5
ENSG00000260122.1	lnc-FIGNL2-6	2.49	0.57	3.86	-0.09	0.031571	chr12	51809705	51810600	-	lincRNA	RP11-923I11.3
ENSG00000274294.1	lnc-LRRC28-2	2.44	0.50	3.62	-0.72	0.044668	chr15	99416584	99417204	+	lincRNA	RP11-20G13.5
ENSG00000275613.1	lnc-MRM1-2	2.43	0.48	3.55	-0.90	0.049025	chr17	36722583	36726340	+	lincRNA	CTD-3194G12.2
ENSG00000274565.1	lnc-MARCH10-4	2.41	0.49	3.55	-0.88	0.048640	chr17	62626437	62627590	-	antisense	CTD-3035K23.7
ENSG00000255089.1	lnc-IFITM2-2	2.39	0.49	3.78	-0.30	0.035607	chr11	321991	322426	+	antisense	RP11-326C3.10
ENSG00000234504.2	TMEM72-AS1	2.35	0.45	3.55	-0.90	0.049025	chr10	44899614	44920220	-	antisense	RP11-285G1.2
ENSG00000275223.1	lnc-NECAB3-2	2.30	0.42	3.63	-0.70	0.044196	chr20	33655701	33656423	-	lincRNA	RP1-63M2.7
ENSG00000232021.6	LEF1-AS1	-2.37	5.33	-3.83	-0.28	0.032327	chr4	108167525	108256836	+	processed_transcript	LEF1-AS1
ENSG00000256039.1	LINC02446	-2.56	6.51	-3.91	-0.28	0.028352	chr12	10553363	10558049	+	lincRNA	RP11-291B21.2
ENSG00000260940.1	lnc-S1PR1-1	-2.65	4.37	-3.86	-0.13	0.031571	chr1	101243158	101243749	+	sense_overlapping	RP4-575N6.5
ENSG00000205056.8	LINC02397	-2.78	5.21	-3.62	-0.87	0.044284	chr12	92466451	92492091	+	lincRNA	RP11-693J15.5
ENSG00000272909.1	lnc-ZBTB25-1	-2.81	3.67	-3.78	-0.29	0.035607	chr14	64440369	64442238	-	antisense	CTD-2555O16.4
ENSG00000187621.14	TCL6	-2.85	6.37	-3.78	-0.64	0.035607	chr14	95650498	95679833	+	processed_transcript	TCL6
ENSG00000267521.1	lnc-SRSF2-2	-2.91	4.54	-3.77	-0.38	0.035780	chr17	76957023	76958222	-	lincRNA	RP11-87G24.6
ENSG00000273888.1	FRMD6-AS1	-2.91	2.46	-3.78	-0.26	0.035607	chr14	51649516	51651744	-	antisense	FRMD6-AS1
ENSG00000233355.6	CHRM3-AS2	-3.11	6.70	-6.40	6.92	0.000283	chr1	239703381	239730465	-	antisense	CHRM3-AS2
ENSG00000245164.6	LINC00861	-3.15	7.08	-6.93	8.43	0.000188	chr8	125922308	125951249	-	lincRNA	LINC00861
ENSG00000224020.1	MIR181A2HG	-3.18	3.29	-3.75	-0.37	0.037235	chr9	124658467	124698631	+	antisense	MIR181A2HG
ENSG00000277301.1	/	-3.19	3.39	-4.67	2.08	0.006709	chr20	32509959	32520285	+	antisense	RP5-1184F4.7
ENSG00000260711.2	lnc-TC2N-1	-3.21	4.05	-3.84	-0.18	0.031861	chr14	91752856	91759798	-	sense_intronic	RP11-747H7.3
ENSG00000235532.1	LINC00402	-3.21	4.71	-5.18	3.50	0.002517	chr13	74231457	74259976	+	lincRNA	LINC00402
ENSG00000273018.5	/	-3.21	5.39	-4.51	1.54	0.008557	chr17	18511221	18551705	-	processed_transcript	CTD-2303H24.2
ENSG00000249655.1	lnc-ANKRD34B-1	-3.22	1.87	-3.67	-0.54	0.041025	chr5	80630313	80631590	-	antisense	CTC-325J23.2
ENSG00000224259.6	LINC01133	-3.24	4.21	-3.95	0.09	0.026526	chr1	159961218	159984750	+	lincRNA	LINC01133
ENSG00000267074.1	lnc-SLFN14-3	-3.25	4.29	-4.23	0.86	0.014507	chr17	35499690	35510270	-	sense_intronic	RP11-1094M14.5
ENSG00000223855.1	lnc-DNAAF5-3	-3.26	1.82	-3.61	-0.69	0.045094	chr7	520391	525232	+	lincRNA	HRAT92
ENSG00000275963.1	lnc-CREBL2-4	-3.30	1.67	-3.69	-0.49	0.039782	chr12	12648939	12649713	+	lincRNA	RP11-180M15.6
ENSG00000235185.1	lnc-TMCO4-1	-3.33	0.72	-3.58	-0.76	0.046524	chr1	19591802	19596832	-	antisense	RP5-1056L3.1
ENSG00000246223.8	LINC01550	-3.35	4.91	-5.11	3.29	0.002857	chr14	97925610	97978124	-	lincRNA	LINC01550
ENSG00000271392.1	lnc-GAS2L2-4	-3.35	0.45	-3.55	-0.83	0.048640	chr17	35757199	35758325	-	sense_intronic	RP1-161P9.5
ENSG00000264745.1	TTC39C-AS1	-3.40	1.26	-3.71	-0.43	0.039067	chr18	23994213	24015339	-	antisense	TTC39C-AS1
ENSG00000270951.1	lnc-CTSZ-2	-3.53	-0.26	-3.56	-0.83	0.048252	chr20	58876592	58876981	-	antisense	RP1-309F20.4
ENSG00000259407.1	lnc-KLHL25-1	-3.54	0.92	-3.75	-0.34	0.037090	chr15	85744109	85750281	-	antisense	RP11-158M2.3
ENSG00000267568.6	lnc-SRSF2-2	-3.57	3.77	-3.86	-0.13	0.031571	chr17	76950317	76969156	-	processed_transcript	RP11-87G24.3
ENSG00000235576.1	LINC01871	-3.59	2.10	-4.04	0.40	0.021998	chr2	7725801	7730705	+	lincRNA	AC092580.4
ENSG00000236304.1	#N/B	-3.60	1.69	-3.70	-0.47	0.039592	chr11	76657056	76663866	+	antisense	AP001189.4
ENSG00000255325.2	lnc-DERL1-7	-3.69	1.46	-3.64	-0.62	0.043649	chr8	122485515	122489815	-	lincRNA	RP11-96B2.1
ENSG00000235304.1	LINC01281	-3.70	1.29	-4.24	0.90	0.014333	chrX	39304956	39327362	-	lincRNA	LINC01281
ENSG00000250790.4	lnc-ZNF26-12	-3.71	0.95	-3.59	-0.73	0.046068	chr12	132911470	132914732	+	lincRNA	RP11-46H11.3
ENSG00000250334.5	LINC00989	-3.78	3.17	-3.69	-0.51	0.039592	chr4	79492416	79576460	+	lincRNA	LINC00989
ENSG00000241657.1	lnc-MGAM2-1	-3.80	1.67	-3.86	-0.05	0.031571	chr7	142433895	142434394	+	lincRNA	TRBV11-2
ENSG00000225948.2	PRKCQ-AS1	-3.83	2.91	-4.81	2.48	0.004939	chr10	6618716	6625346	+	lincRNA	RP11-554I8.1
ENSG00000247498.9	GPRC5D-AS1	-3.86	1.53	-4.06	0.45	0.021259	chr12	12927726	12984645	+	antisense	RP11-392P7.6
ENSG00000267239.1	lnc-PTPN2-3	-3.93	0.29	-4.13	0.60	0.018010	chr18	13203774	13216367	-	antisense	RP11-794M8.1
ENSG00000278029.1	/	-3.99	1.74	-4.45	1.48	0.009738	chr15	81392759	81393384	+	lincRNA	CTD-2240J17.2
ENSG00000235728.1	lnc-ANLN-3	-4.01	2.12	-4.54	1.71	0.008328	chr7	36781008	36782789	+	lincRNA	AC007349.5
ENSG00000273348.1	lnc-CTAGE1-6	-4.08	-0.14	-4.42	1.34	0.010303	chr18	22347846	22348252	-	lincRNA	RP11-535A5.1
ENSG00000230910.2	lnc-LMBRD1-5	-4.08	0.51	-3.95	0.15	0.026526	chr6	68629962	68635027	-	antisense	RP3-525N10.2
ENSG00000237410.1	lnc-SLC22A12-2	-4.14	0.97	-4.10	0.55	0.019497	chr11	64646399	64659681	+	antisense	AP001092.4
ENSG00000254968.6	lnc-PTS-3	-4.14	0.47	-4.70	2.10	0.006235	chr11	112393118	112621729	+	lincRNA	RP11-65M17.3
ENSG00000249669.8	/	-4.17	0.49	-4.57	1.76	0.007952	chr5	149406689	149432835	+	lincRNA	CARMN
ENSG00000250390.2	lnc-CEP57-2	-4.24	0.94	-4.55	1.72	0.008121	chr11	95482406	95497553	+	lincRNA	RP11-338H14.1
ENSG00000224050.1	lnc-RFPL3-2	-4.46	1.31	-4.88	2.62	0.004410	chr22	32273420	32277186	+	lincRNA	RP1-90G24.6
ENSG00000276778.1	LINC02227	-4.48	0.40	-4.87	2.55	0.004410	chr5	158320683	158409773	-	lincRNA	RP11-524N5.1
ENSG00000263667.1	lnc-EXOC2-6	-4.51	1.14	-4.52	1.66	0.008482	chr6	181466	205484	-	lincRNA	RP3-416J7.4
ENSG00000242082.1	SLC5A4-AS1	-4.66	0.20	-5.04	3.01	0.003279	chr22	32205115	32269666	+	antisense	RP1-90G24.10
ENSG00000259986.1	lnc-SH3GL3-5	-4.67	2.45	-4.91	2.75	0.004119	chr15	83179182	83439445	+	antisense	RP11-382A20.4
ENSG00000269600.1	lnc-MZF1-1	-4.67	2.58	-5.80	5.22	0.000697	chr19	58593896	58599355	-	antisense	AC016629.3
ENSG00000272240.1	lnc-OR4F21-2	-4.72	1.72	-4.76	2.34	0.005454	chr8	234347	234887	-	antisense	RP5-855D21.1
ENSG00000249667.1	LINC01259	-4.78	2.10	-5.95	5.61	0.000522	chr4	38509767	38518056	+	lincRNA	LINC01259
ENSG00000267128.1	lnc-ZACN-64	-5.27	0.79	-6.76	7.76	0.000236	chr17	76140556	76154650	+	antisense	RP11-449J21.5

Table S4. Differentially expressed lncRNAs in molecular subgroups vs PNBM using EdgeR

Abbreviations: lncRNA = long non-coding RNA; JMML = juvenile myelomonocytic leukemia; PNBM = pediatric normal bone marrow; FC = fold change; adj.P = adjusted P-value

KRAS vs PN samples

Ensembl_gene	LNCipedia_name	logFC	AveExpr	t	P.Value	adj.P.Val	B	Symbol
ENSG00000224050.1	lnc-RFPL3-2	-6.133056989	1.79585239	-6.895718811	1.13598E-07	0.000972283	7.476489045	RP1-90G24.6
ENSG00000233785.1	lnc-ACOT9-1	5.56826821	3.377442335	6.396319533	4.49371E-07	0.001523052	6.039862547	RP13-314C10.5
ENSG00000259986.1	lnc-SH3GL3-5	-6.733064669	2.935464639	-6.28411095	6.13931E-07	0.001523052	5.984835628	RP11-382A20.4
ENSG00000263050.1	lnc-SMG6-2	6.206114971	4.175964552	6.23105031	7.1179E-07	0.001523052	5.663126358	RP11-667K14.3
ENSG00000248884.1	lnc-CCDC125-6	5.736698725	1.814558371	5.87826422	1.91246E-06	0.003117363	4.795942406	CTC-537E7.3
ENSG00000274092.1	lnc-NSMCE1-11	5.590795628	2.072140008	5.812689541	2.30007E-06	0.003117363	4.616917648	CTD-3203P2.3
ENSG00000276778.1	LINC02227	-5.189949942	0.893287799	-5.769481643	2.5978E-06	0.003117363	4.567981767	RP11-524N5.1
ENSG00000237410.1	lnc-SLC22A12-2	-5.488755857	1.455910717	-5.711933189	3.05546E-06	0.003117363	4.449126179	AP001092.4
ENSG00000223662.1	/	6.106553601	3.145812525	5.687016985	3.27798E-06	0.003117363	4.382882979	SAMSN1-AS1
ENSG00000261573.1	lnc-ATP6V1G3-2	5.571073271	1.646552741	5.605561682	4.12567E-06	0.003531162	4.115079487	RP11-553K8.5
ENSG00000242082.1	SLC5A4-AS1	-5.256603196	0.686476165	-5.5580848	4.71807E-06	0.003671089	4.037122008	RP1-90G24.10
ENSG00000228221.5	LINC00578	5.215127158	1.624319628	5.406120426	7.25229E-06	0.005172694	3.576925092	LINC00578
ENSG00000254968.6	lnc-PTS-3	-4.664740732	0.957319971	-5.333010316	8.92038E-06	0.005873043	3.415743073	RP11-65M17.3
ENSG00000259999.1	lnc-BCAR1-1	4.994799264	2.560182004	5.211720372	1.25779E-05	0.007689616	3.081526921	RP11-252K23.1
ENSG00000215304.3	lnc-GOLGA8K-1	5.331800903	3.289685744	5.146784391	1.51188E-05	0.008626771	2.958366338	RP13-395E19.3
ENSG00000269600.1	lnc-MZF1-1	-6.013269961	3.070294095	-4.903091562	3.0148E-05	0.016127288	2.344523643	AC016629.3
ENSG00000254602.1	lnc-CLP1-3	-4.683813025	3.273269936	-4.628219036	6.55343E-05	0.031343313	1.60344775	AP000662.4
ENSG00000267503.1	lnc-FAM210A-3	5.448745003	1.649707469	4.626155501	6.59165E-05	0.031343313	1.645223466	RP11-53B2.4
ENSG00000226644.5	lnc-STK35-4	5.715080463	3.501221223	4.581892955	7.4669E-05	0.033636429	1.554292981	RP11-128M1.1
ENSG00000236753.5	MKLN1-AS	4.92636704	4.342386467	4.558485804	7.9754E-05	0.034130716	1.338337762	MKLN1-AS
ENSG00000224969.1	lnc-HES4-1	5.09660141	1.352245209	4.537889104	8.45115E-05	0.03444446	1.432904107	RP11-54O7.11
ENSG00000228315.11	lnc-IGLL1-1	4.259393085	2.119259742	4.502900566	9.32463E-05	0.035790366	1.311799832	GUSBP11
ENSG00000259407.1	lnc-KLHL25-1	-4.443902544	1.406178018	-4.491885985	9.61769E-05	0.035790366	1.335259293	RP11-158M2.3
ENSG00000257747.1	LINC02426	4.827269926	0.748397878	4.473145892	0.000101374	0.036152535	1.261429938	RP11-362A1.1
ENSG00000247775.2	SNCA-AS1	-4.77426678	2.489246419	-4.434412722	0.000113013	0.038691167	1.199397493	SNCA-AS1
ENSG00000248719.1	lnc-ANTXR2-1	4.38415308	2.827330238	4.32004784	0.000155657	0.047840906	0.873409911	RP11-377G16.2
ENSG00000243179.1	LINC01191	5.570744024	2.911662543	4.312124638	0.00015914	0.047840906	0.906967561	AC110769.3
ENSG00000255801.1	lnc-CLEC4E-1	6.052706647	3.872496476	4.307672746	0.000161131	0.047840906	0.906242736	RP11-561P12.5
ENSG00000264812.1	lnc-C17orf62-3	4.627971165	2.232696845	4.305533945	0.000162097	0.047840906	0.83086653	RP13-20L14.4

NRAS vs PN samples

Ensembl_gene	LNCipedia_name	logFC	AveExpr	t	P.Value	adj.P.Val	B	Symbol
ENSG00000253619.1	lnc-MTBP-2	5.9779136	2.445657706	7.535057087	2.02835E-08	0.000103186	8.929262522	RP11-369K17.1
ENSG00000263050.1	lnc-SMG6-2	6.809022273	4.175964552	7.47011302	2.41117E-08	0.000103186	8.770974849	RP11-667K14.3
ENSG00000249662.5	LINC02218	5.934663991	2.767722071	6.533437888	3.07347E-07	0.000791069	6.525084178	RP11-321E2.4
ENSG00000254983.1	lnc-PARVA-7	5.706895118	2.753363318	6.416827284	4.24508E-07	0.000791069	6.216677086	RP11-573E11.2
ENSG00000233785.1	lnc-ACOT9-1	5.378163047	3.377442335	6.37863413	4.71991E-07	0.000791069	6.063855138	RP13-314C10.5
ENSG00000215304.3	lnc-GOLGA8K-1	5.959263242	3.289685744	6.320650162	5.54553E-07	0.000791069	5.967886848	RP13-395E19.3
ENSG00000256571.1	lnc-RXYLT1-1	5.288265602	1.956564176	6.139585806	9.18942E-07	0.001123603	5.473621719	RP11-274J7.2
ENSG00000233554.5	/	5.251084351	2.24108038	5.897457668	1.81197E-06	0.001766046	4.875891408	B4GALT1-AS1
ENSG00000250508.1	lnc-IGHMBP2-1	5.261214373	1.482153948	5.888720278	1.85704E-06	0.001766046	4.713177059	RP11-757G1.6
ENSG00000274092.1	lnc-NSMCE1-11	5.111626246	2.072140008	5.382369015	7.75674E-06	0.005841723	3.579820319	CTD-3203P2.3
ENSG00000248719.1	lnc-ANTXR2-1	4.91679934	2.827330238	5.364047646	8.16977E-06	0.005841723	3.506629421	RP11-377G16.2
ENSG00000278727.1	lnc-ACOD1-5	4.927021061	2.039380164	5.336961056	8.82113E-06	0.005841723	3.457479547	AC000403.4
ENSG00000257696.1	lnc-ACTR6-1	4.971605049	1.780974324	5.334898247	8.87281E-06	0.005841723	3.456067488	RP11-175P13.2
ENSG00000233061.1	TTLL7-IT1	5.764416803	2.732026234	5.20663127	1.27606E-05	0.007356494	3.187742601	TTLL7-IT1
ENSG00000249669.8	/	-4.772101069	0.981459147	-5.203000982	1.28926E-05	0.007356494	3.144012154	CARMN
ENSG00000231842.1	lnc-TRMT11-2	4.712821181	2.256386076	5.123119001	1.61673E-05	0.008648494	2.912278647	RP11-527F13.1
ENSG00000267128.1	lnc-ZACN-64	-4.970067732	1.27642637	-5.061185248	1.92682E-05	0.00970099	2.751064952	RP11-449J21.5
ENSG00000232243.1	lnc-TUBA3C-1	4.786175907	0.440678049	5.016081535	2.18941E-05	0.01009509	2.648360165	RP11-408E5.5
ENSG00000242082.1	SLC5A4-AS1	-4.717875526	0.686476165	-4.997812687	2.30568E-05	0.01009509	2.639421188	RP1-90G24.10
ENSG00000230753.5	ZNF341-AS1	5.210075132	1.951098039	4.981955716	2.41157E-05	0.01009509	2.590255833	ZNF341-AS1
ENSG00000254968.6	lnc-PTS-3	-4.40628501	0.957319971	-4.9725179	2.47689E-05	0.01009509	2.526092742	RP11-65M17.3
ENSG00000235304.1	LINC01281	-4.680699914	1.779032863	-4.938958329	2.72377E-05	0.010596699	2.452674692	LINC01281
ENSG00000259473.1	LINC02205	4.675534462	1.818430864	4.835828956	3.64667E-05	0.013570357	2.196097643	RP11-96C21.1
ENSG00000229005.2	HNF4A-AS1	-4.644423478	1.1939965	-4.803738578	3.993E-05	0.014240021	2.135870228	HNF4A-AS1
ENSG00000261916.1	lnc-SHPK-1	4.753348081	1.912821856	4.706031764	5.26209E-05	0.01801529	1.876040773	RP11-235E17.4
ENSG00000246363.2	LINC02458	6.055009835	2.856946072	4.684213609	5.59625E-05	0.018247688	1.797948612	RP11-13A1.1
ENSG00000273348.1	lnc-CTAGE1-6	-4.346425888	0.349529447	-4.674213521	5.75637E-05	0.018247688	1.794203257	RP11-535A5.1
ENSG00000226644.5	lnc-STK35-4	5.550295592	3.501221223	4.574417417	7.6257E-05	0.022944511	1.553958124	RP11-128M1.1
ENSG00000225329.3	LHFPL3-AS2	4.274251382	1.93405681	4.567566994	7.77417E-05	0.022944511	1.518594899	LHFPL3-AS2
ENSG00000259376.1	lnc-CHSY1-3	4.375161681	0.894851412	4.531182968	8.61378E-05	0.024570141	1.431726135	RP11-505E24.3
ENSG00000228315.11	lnc-IGLL1-1	3.993939588	2.119259742	4.482010786	9.88818E-05	0.027300957	1.302405528	GUSBP11
ENSG00000226004.1	lnc-PERP-1	4.602536962	1.278366199	4.450828097	0.000107927	0.028099437	1.238979455	RP11-10J5.1
ENSG00000253616.5	lnc-TNFRSF10B-1	5.373104354	2.694390489	4.449467665	0.00010834	0.028099437	1.228101516	RP11-875O11.3
ENSG00000240893.1	LINC02042	4.629135993	0.541919427	4.434869857	0.000112868	0.028412939	1.202227874	RP11-572C15.5
ENSG00000257156.1	LINC02458	5.409813487	1.668535841	4.41153499	0.000120499	0.029467152	1.162346668	RP11-13A1.3
ENSG00000237741.1	lnc-IDS-3	4.461680216	0.847201839	4.389525115	0.000128163	0.030470728	1.085869522	AC002368.4
ENSG00000258760.1	lnc-MAX-2	4.588421863	1.41819875	4.367292646	0.000136393	0.031551093	1.03449232	CTD-2509G16.5
ENSG00000270190.1	lnc-ANAPC1-5	4.136740144	1.809993757	4.336444747	0.000148684	0.033489223	0.94903403	RP11-803D5.4
ENSG00000228022.5	HCG20	4.859666684	2.055767261	4.286486465	0.000170949	0.035778116	0.841603648	HCG20
ENSG00000203801.8	LINC00222	4.798117019	2.003933502	4.28104898	0.000173563	0.035778116	0.826890667	LINC00222
ENSG00000248884.1	lnc-CCDC125-6	4.426195685	1.814558371	4.279725473	0.000174205	0.035778116	0.815260969	CTC-537E7.3
ENSG00000236866.5	lnc-GDAP2-1	3.691805477	0.775487609	4.276932215	0.000175567	0.035778116	0.799125359	AL157902.3
ENSG00000255314.2	lnc-CREB3L1-2	4.245582132	1.213876828	4.251979081	0.000188217	0.037464032	0.745751671	RP11-702F3.3
ENSG00000259999.1	lnc-BCAR1-1	4.050291902	2.560182004	4.229142359	0.00020058	0.037630793	0.686645664	RP11-252K23.1
ENSG00000256862.1	lnc-PRMT8-2	-4.440368324	2.191508078	-4.227108028	0.000201719	0.037630793	0.686729584	RP11-664D1.1
ENSG00000230257.1	/	5.520118645	3.047830506	4.222226935	0.000204479	0.037630793	0.691642472	NFE4
ENSG00000204528.3	PSORS1C3	4.119533843	1.177301065	4.210958459	0.000210993	0.037630793	0.643667473	PSORS1C3
ENSG00000263603.1	lnc-ATAD5-2	4.40567422	2.660170693	4.210881075	0.000211038	0.037630793	0.648503954	CTD-2349P21.5
ENSG00000245164.6	LINC00861	-3.049798976	7.569837542	-4.198054733	0.000218704	0.038201721	0.401853373	LINC00861
ENSG00000276778.1	LINC02227	-4.281140745	0.893287799	-4.160987115	0.00024243	0.041499208	0.515527146	RP11-524N5.1
ENSG00000254826.1	lnc-UVRAG-1	5.793481025	5.245153429	4.132152108	0.000262623	0.043262033	0.372084255	CTD-2530H12.2
ENSG00000236753.5	MKLN1-AS	4.433876324	4.342386467	4.131857679	0.000262837	0.043262033	0.369988785	MKLN1-AS

ENSG00000271133.5	lnc-MACC1-3	4.599796487	2.254998236	4.117470669	0.000273531	0.044172641	0.422668951	CTA-293F17.1
ENSG00000267834.1	lnc-SH3GLB2-1	3.873402876	0.723671344	4.095753108	0.000290488	0.044584466	0.36222499	RP11-167N5.5
ENSG00000263812.5	LINC00908	3.8282902	0.556600205	4.095228398	0.00029091	0.044584466	0.360640297	LINC00908
ENSG00000227938.1	lnc-TRMT61B-3	4.694216922	1.871476208	4.094238322	0.000291708	0.044584466	0.36822624	AC104695.4
ENSG00000273018.5	/	-5.38163684	5.883656358	-4.07607805	0.000306739	0.045862234	0.329532425	CTD-2303H24.2
ENSG00000253532.1	lnc-OSR2-2	4.233592293	3.559107054	4.071338873	0.000310785	0.045862234	0.271014532	CTD-2340D6.1
ENSG00000231729.1	ARHGEF9-IT1	4.340093202	1.279894934	4.057495006	0.000322907	0.046843396	0.274304878	ARHGEF9-IT1
ENSG00000237845.1	lnc-NID1-2	4.385375051	1.425187349	4.035434385	0.000343189	0.048955927	0.221952057	RP5-940F7.2
ENSG00000272240.1	lnc-OR4F21-2	-5.37936089	2.213170537	-4.023408627	0.000354767	0.049777835	0.196620408	RP5-855D21.1

NF1 vs PN samples

Ensembl_gene	LNCipedia_name	logFC	AveExpr	t	P.Value	adj.P.Val	B	Symbol
ENSG00000255571.7	/	-6.976968938	4.218722612	-8.297453205	2.76978E-09	1.1143E-05	10.72225245	MIR9-3HG
ENSG00000225948.2	PRKCQ-AS1	-7.307860721	3.3961034	-8.165165516	3.89236E-09	1.1143E-05	10.51009374	RP11-554I8.1
ENSG00000258512.1	LINC00239	-6.267408204	4.092360709	-8.163839042	3.90571E-09	1.1143E-05	10.28924832	LINC00239
ENSG00000249662.5	LINC02218	7.212044263	2.767722071	7.022256425	8.04883E-08	0.000172225	7.55722972	RP11-321E2.4
ENSG00000259986.1	lnc-SH3GL3-5	-7.554941403	2.935464639	-6.828933745	1.36344E-07	0.000233394	7.35988484	RP11-382A20.4
ENSG00000278029.1	/	-6.314810179	2.227756401	-6.33371431	5.3476E-07	0.000762836	6.024611597	CTD-2240J17.2
ENSG00000249655.1	lnc-ANKRD34B-1	-5.761090537	2.363768074	-6.163128563	8.60415E-07	0.001052042	5.536247653	CTC-325J23.2
ENSG00000259418.1	lnc-DUOX2-2	6.954309652	1.474624971	6.042953266	1.2044E-06	0.001288558	5.172262329	RP11-109D20.1
ENSG00000276278.1	lnc-WDR73-9	-5.861692928	4.240898757	-5.972050802	1.46941E-06	0.001309463	5.073121363	RP11-182J1.3
ENSG00000228109.1	MELTF-AS1	-5.428379184	3.096501865	-5.957675095	1.52992E-06	0.001309463	4.983971048	MFI2-AS1
ENSG00000272240.1	lnc-OR4F21-2	-6.877269054	2.213170537	-5.872351555	1.94453E-06	0.001513022	4.918129641	RP5-855D21.1
ENSG00000258824.2	lnc-ZBTB25-2	-5.574734458	3.783606315	-5.795450668	2.41451E-06	0.001722148	4.603348048	CTD-2555O16.2
ENSG00000261643.1	lnc-LRPAP1-1	-5.047885449	4.262733086	-5.701764742	3.14438E-06	0.001871795	4.298311356	RP11-529E10.6
ENSG00000247498.9	GPRC5D-AS1	-5.95788468	2.021783749	-5.669057262	3.44842E-06	0.001871795	4.338481242	RP11-392P7.6
ENSG00000250390.2	lnc-CEP57-2	-5.798248495	1.431276471	-5.661233911	3.52543E-06	0.001871795	4.2954088	RP11-338H14.1
ENSG00000260807.6	lnc-LMF1-3	-6.011689185	2.484921961	-5.648612363	3.65332E-06	0.001871795	4.279066092	RP11-161M6.2
ENSG00000272733.1	lnc-IGLL1-1	-5.694129936	3.548033036	-5.642417777	3.71779E-06	0.001871795	4.245502412	KB-208E9.1
ENSG00000247199.3	lnc-HTR4-1	-5.464404345	3.137001948	-5.602978019	4.1559E-06	0.00191303	4.128395997	RP11-373N22.3
ENSG00000254968.6	lnc-PTS-3	-5.136890977	0.957319971	-5.595327581	4.24671E-06	0.00191303	4.060367512	RP11-65M17.3
ENSG00000227678.7	lnc-SAMD3-1	-5.885859117	3.31324121	-5.569170896	4.57252E-06	0.001956808	4.075215325	RP11-73O6.3
ENSG00000272663.1	lnc-LHCGR-2	-5.385437755	2.019681102	-5.458370575	6.2553E-06	0.002549482	3.757324348	RP11-191L17.1
ENSG00000251093.1	lnc-ARRDC3-4	6.264230874	0.685925767	5.384448738	7.7112E-06	0.002776672	3.457074632	RP11-414H23.3
ENSG00000255801.1	lnc-CLEC4E-1	7.780072168	3.872496476	5.38338048	7.73455E-06	0.002776672	3.636499499	RP11-561P12.5
ENSG00000276778.1	LINC02227	-5.336836902	0.893287799	-5.378819468	7.83509E-06	0.002776672	3.552596352	RP11-524N5.1
ENSG00000263667.1	lnc-EXOC2-6	-6.097783029	1.626702939	-5.366623836	8.11039E-06	0.002776672	3.584383694	RP3-416J7.4
ENSG00000267327.1	lnc-WDR7-6	5.83273721	0.267846154	5.342780556	8.67693E-06	0.002856378	3.277404372	CTD-2008L17.1
ENSG00000267239.1	lnc-PTPN2-3	-4.760883571	0.780238476	-5.312845536	9.4447E-06	0.002973966	3.325156249	RP11-794M8.1
ENSG00000237410.1	lnc-SLC22A12-2	-5.635642817	1.455910717	-5.290386722	1.00651E-05	0.002973966	3.350842865	AP001092.4
ENSG00000235978.6	EGOT	-5.618916294	3.376136932	-5.289985395	1.00765E-05	0.002973966	3.347454559	AC018816.3
ENSG00000250131.1	lnc-NEIL3-6	-4.812017647	3.398975457	-5.273206456	1.05671E-05	0.003014783	3.22628492	RP11-130F10.1
ENSG00000233785.1	lnc-ACOT9-1	5.745029684	3.377442335	5.240597979	1.15898E-05	0.003187646	3.019169323	RP13-314C10.5
ENSG00000251381.7	LINC00958	7.13368989	2.730066504	5.221796725	1.22239E-05	0.003187646	3.059983042	LINC00958
ENSG00000249669.8	/	-5.266368833	0.981459147	-5.219886612	1.22903E-05	0.003187646	3.141004724	CARMN
ENSG00000236204.5	LINC01376	-4.76394017	3.636375427	-5.112993502	1.66378E-05	0.00418833	2.84327714	LINC01376
ENSG00000255325.2	lnc-DERL1-7	-5.70981425	1.94817527	-5.091085789	1.77033E-05	0.004320974	2.863470136	RP11-96B2.1
ENSG00000267583.5	lnc-INO80C-1	-5.39458635	2.563145612	-5.081814209	1.81744E-05	0.004320974	2.814742914	RP11-322E11.5
ENSG00000245869.2	lnc-BCL9L-1	-6.153224801	3.242443127	-5.031521787	2.09573E-05	0.004847928	2.731792526	RP11-158I9.5
ENSG00000251095.6	lnc-MMRN1-12	5.769758027	0.360963028	5.015306967	2.19422E-05	0.004942197	2.492241488	RP11-115D19.1
ENSG00000235785.1	lnc-CCNK-1	-5.067584352	2.020682054	-4.970304904	2.49246E-05	0.005469986	2.518226047	AL109767.1
ENSG00000261291.1	lnc-RBL2-2	-4.724466224	4.384724225	-4.95219277	2.62361E-05	0.005613864	2.438765177	RP11-295M3.2
ENSG00000250012.1	lnc-ROPN1B-2	-5.381546564	1.902159034	-4.91095283	2.94847E-05	0.006065206	2.386416333	RP11-124N2.1
ENSG00000228857.2	lnc-SLC35F5-3	-5.174391332	3.052283156	-4.907636928	2.97627E-05	0.006065206	2.370755009	AC104653.1
ENSG00000249309.1	lnc-TLR2-1	6.18356924	4.254939727	4.758199338	4.54135E-05	0.009039409	1.810486476	RP11-153M7.5
ENSG00000235185.1	lnc-TMCO4-1	-4.738527188	1.211189741	-4.716808745	5.10443E-05	0.009909885	1.868036527	RP5-1056L3.1
ENSG00000272555.1	lnc-CYP27A1-1	5.875928025	1.19789154	4.706545673	5.25446E-05	0.009909885	1.772573971	RP11-459I19.1
ENSG00000276718.1	lnc-TAPBPL-3	-5.430116485	3.022294789	-4.701752477	5.32603E-05	0.009909885	1.87542727	RP1-102E24.10
ENSG00000249572.1	lnc-ADAMTS12-6	-5.433657856	4.14323283	-4.689530261	5.51293E-05	0.009946422	1.698755697	CTD-2203K17.1
ENSG00000255364.1	SMILR	-5.532540497	1.69866789	-4.685365775	5.57808E-05	0.009946422	1.841671876	RP11-94A24.1
ENSG00000257696.1	lnc-ACTR6-1	5.593701632	1.780974324	4.669764522	5.82906E-05	0.010022968	1.646977731	RP11-175P13.2
ENSG00000227189.2	lnc-FGFRL1-9	-4.967849142	1.880292545	-4.66817689	5.85522E-05	0.010022968	1.758598902	AC092535.3
ENSG00000236753.5	MKLN1-AS	5.488002846	4.342386467	4.658313913	6.02037E-05	0.010103603	1.524631682	MKLN1-AS
ENSG00000260350.1	lnc-CARHSP1-1	-5.566181932	3.196983556	-4.647145815	6.21297E-05	0.010226309	1.737943731	RP11-152P23.2
ENSG00000240240.8	lnc-ANKRD20A2-16	-5.133005855	3.060972467	-4.613483598	6.83127E-05	0.010995886	1.634351907	RP11-146D12.2
ENSG00000248489.1	LINC02062	-4.851104713	2.157397885	-4.608007559	6.93747E-05	0.010995886	1.608731633	CTD-2007H13.3
ENSG00000197182.13	/	-5.735184685	3.241964265	-4.588927851	7.32046E-05	0.011391964	1.599939955	MIRLET7BHG
ENSG00000203999.8	LINC01270	6.435202897	4.230856393	4.568522675	7.75328E-05	0.011850063	1.389821494	LINC01270
ENSG00000273893.1	lnc-TP53RK-6	-4.776654031	3.399110792	-4.559204447	7.95929E-05	0.011951498	1.48947743	RP1-101A2.1
ENSG00000256101.5	lnc-CLEC6A-1	-4.524555749	1.800395557	-4.546105709	8.25808E-05	0.012186356	1.431613576	RP11-90D4.3
ENSG00000233308.1	OSTN-AS1	5.401988014	0.377680538	4.533959214	8.54507E-05	0.012333458	1.301613518	OSTN-AS1
ENSG00000259372.2	/	-5.197387693	2.502184597	-4.528897904	8.66755E-05	0.012333458	1.432340559	CTD-2240J17.1
ENSG00000228203.6	RNF144A-AS1	-4.578892672	2.311651441	-4.5201339	8.88377E-05	0.012333458	1.372115246	RNF144A-AS1
ENSG00000280207.1	lnc-FAM153C-5	-4.936222178	1.494174037	-4.518121798	8.93416E-05	0.012333458	1.389623889	RP11-423H2.4
ENSG00000229005.2	HNF4A-AS1	-4.861526948	1.1939965	-4.483956439	9.8343E-05	0.013298228	1.307017381	HNF4A-AS1
ENSG00000255487.1	/	-4.532292644	2.531852909	-4.48001486	9.94376E-05	0.013298228	1.272237935	RP11-513D5.5
ENSG00000253134.1	lnc-LSM11-4	-6.173586149	2.68438111	-4.46476572	0.000103787	0.013604986	1.301937284	CTC-436K13.2
ENSG00000180422.3	LINC00304	-5.208905552	3.162566435	-4.460930348	0.000104911	0.013604986	1.259763576	LINC00304
ENSG00000238273.3	lnc-C2orf49-2	-4.98703478	1.841604947	-4.451619432	0.000107688	0.013756716	1.232540763	AC012360.6
ENSG00000276255.2	lnc-C1orf35-3	-4.898732536	2.651871414	-4.429601307	0.000114548	0.014257066	1.173968761	RP5-881P19.7
ENSG00000269680.1	lnc-SH2D3A-2	-5.038001167	1.75441769	-4.428396003	0.000114936	0.014257066	1.173451063	CTD-3128G10.6
ENSG00000275910.1	lnc-NTAN1-4	-4.748714248	2.641731243	-4.422421576	0.000116877	0.014290776	1.150056327	RP11-680G24.6
ENSG00000273348.1	lnc-CTAGE1-6	-4.563529358	0.349529447	-4.411924996	0.000120367	0.014309698	1.115969049	RP11-535A5.1
ENSG00000229956.10	ZRANB2-AS2	-4.228887126	3.119376011	-4.411899159	0.000120376	0.014309698	1.090066719	ZRANB2-AS2
ENSG00000258168.5	lnc-KCNMB4-1	-4.998377209	2.217997374	-4.389245491	0.000128263	0.015038433	1.080064152	RP11-588H23.3
ENSG00000254362.1	lnc-PPP2R2A-2	-4.71936581	2.335705447	-4.379730767	0.000131726	0.015235764	1.041802573	RP11-14I17.3
ENSG00000253532.1	lnc-OSR2-2	5.220766922	3.559107054	4.327241411	0.000152559	0.017410029	0.789057081	CTD-2340D6.1
ENSG00000233387.1	lnc-ITGB1-6	-4.827609884	1.327253915	-4.295877764	0.000166526	0.01853736	0.843817949	RP11-342D11.3

ENSG00000225637.1	lnc-CRYAA-2	-4.891063598	2.417804292	-4.29535609	0.000166769	0.01853736	0.841797057	AP001046.6
ENSG00000224034.1	LINC02561	-4.333606484	3.388802616	-4.273419054	0.000177296	0.019283798	0.766670515	RP11-445P17.8
ENSG00000235728.1	lnc-ANLN-3	-5.878776968	2.605272143	-4.272018068	0.00017799	0.019283798	0.734057098	AC007349.5
ENSG00000276131.1	lnc-PRSS54-2	-4.300833076	2.810549011	-4.253045682	0.000187659	0.019829672	0.709254073	RP11-481J2.3
ENSG00000271392.1	lnc-GAS2L2-4	-4.398167459	0.937249191	-4.253038622	0.000187663	0.019829672	0.720550621	RP1-161P9.5
ENSG00000264695.1	lnc-HRH4-1	5.880541974	1.688680731	4.240457915	0.000194357	0.020286654	0.664943683	RP11-178F10.2
ENSG00000266744.1	lnc-CDRT15-3	-4.364366531	1.836665375	-4.235504963	0.000197057	0.02032065	0.677020483	RP11-131K5.1
ENSG00000271970.1	/	4.476763403	-0.11849482	4.226331883	0.000202156	0.020598217	0.488494439	RP3-337H4.10
ENSG00000237836.5	/	-4.711533531	2.222102818	-4.220804701	0.00020529	0.020671508	0.659025451	PHKA2-AS1
ENSG00000268628.2	lnc-CRNKL1-8	5.258449557	1.637092914	4.20375569	0.000215263	0.021423713	0.52045166	RP1-122P22.4
ENSG00000230910.2	lnc-LMBRD1-5	-5.131572735	1.001420974	-4.192525867	0.000222092	0.02184923	0.603098226	RP3-525N10.2
ENSG00000259254.1	lnc-RAD51-1	-5.165669278	3.548223493	-4.172776886	0.000234621	0.022819586	0.55599508	CTD-2339L15.3
ENSG00000272269.1	/	5.330576536	3.887697097	4.152503016	0.000248207	0.023869742	0.351634866	RP11-500C11.3
ENSG00000254983.1	lnc-PARVA-7	5.327291461	2.753363318	4.14472834	0.00025362	0.024119289	0.392919655	RP11-573E11.2
ENSG00000276148.1	lnc-YARS2-4	-4.877604434	3.896737032	-4.123528052	0.000268977	0.025298654	0.348837285	RP11-278C7.4
ENSG00000277440.1	lnc-HMOX2-3	-4.228479725	1.985664089	-4.117094717	0.000273816	0.025473805	0.390678113	RP11-295D4.5
ENSG00000277450.1	lnc-TMEM199-1	-4.728412139	2.375212699	-4.106485984	0.000281982	0.025951459	0.384720112	CTB-96E2.10
ENSG00000266237.1	lnc-PSMA8-1	-5.419027139	4.533531223	-4.094245808	0.000291702	0.026560408	0.302267481	RP11-25D3.1
ENSG00000280953.2	lnc-PTPRE-1	-5.158835179	3.444420753	-4.062521213	0.000318453	0.028690955	0.290449949	LINC01163
ENSG00000259469.1	lnc-FBN1-2	-4.584105257	1.260380174	-4.043322532	0.000335797	0.029641176	0.229885637	RP11-227D13.4
ENSG00000249249.1	lnc-AP3S1-4	-4.645491248	1.937249578	-4.043183195	0.000335926	0.029641176	0.230438769	AC010226.4
ENSG00000235989.3	MORC2-AS1	-4.821451911	2.380260175	-4.039432306	0.000339423	0.029644092	0.224824065	MORC2-AS1
ENSG00000265369.3	PCAT18	-5.330727582	2.252881542	-4.008166428	0.000369993	0.031987548	0.160129689	PCAT18
ENSG00000223662.1	/	5.758532385	3.145812525	3.971008533	0.000409847	0.035078832	0.024873455	SAMSN1-AS1
ENSG00000254826.1	lnc-UVRAG-1	6.000595526	5.245153429	3.941267675	0.000444751	0.037689375	-0.094514591	CTD-2530H12.2
ENSG00000271133.5	lnc-MACC1-3	5.503902116	2.254998236	3.921156391	0.000469987	0.039274064	-0.106664879	CTA-293F17.1
ENSG00000229418.2	lnc-BLNK-3	-4.798585463	1.789789032	-3.919112548	0.000472629	0.039274064	-0.059385536	RP11-35J23.1
ENSG00000218537.1	MIF-AS1	-5.252509713	3.32393816	-3.889724358	0.000512259	0.041795514	-0.127467789	MIF-AS1
ENSG00000250659.2	lnc-ROM1-3	4.048445817	-0.301127634	3.889382744	0.000512739	0.041795514	-0.284985079	RP11-864I4.3
ENSG00000204706.14	MAMDC2-AS1	5.263666141	2.631046229	3.879649426	0.00052658	0.042518823	-0.231684853	MAMDC2-AS1
ENSG00000251152.1	lnc-DRD5-9	-4.596411084	2.611239617	-3.872822547	0.000536505	0.042915388	-0.175276398	RP11-281P23.1
ENSG00000230257.1	/	6.204844792	3.047830506	3.853195727	0.000566069	0.044861002	-0.227837769	NFE4
ENSG00000254981.1	lnc-FGFR1-1	4.944550266	1.127442447	3.843200633	0.000581731	0.04567924	-0.32361997	RP11-350N15.3
ENSG00000261736.1	lnc-IL21R-1	5.219353428	3.154509923	3.838819213	0.000588729	0.045808493	-0.330363325	AC002551.1
ENSG00000270020.1	lnc-MTHFSD-6	-4.298141625	1.940574259	-3.833901899	0.000596681	0.045950416	-0.275371222	RP11-463O9.9
ENSG00000272672.1	lnc-LRRC8D-4	-4.48941632	2.918354674	-3.831080874	0.000601291	0.045950416	-0.276194704	RP11-302M6.5
ENSG00000272862.1	lnc-BEND4-2	5.082527282	1.113562548	3.810303397	0.000636327	0.048197516	-0.393069248	RP11-814H16.2

PTPN11 vs PN samples

Ensembl_gene	LNCipedia_name	logFC	AveExpr	t	P.Value	adj.P.Val	B	Symbol
ENSG00000267128.1	lnc-ZACN-64	-5.940674042	1.27642637	-8.039871574	5.38347E-09	4.60771E-05	10.1978438	RP11-449J21.5
ENSG00000233785.1	lnc-ACOT9-1	4.832879541	3.377442335	6.497647823	3.39329E-07	0.001452161	6.407912143	RP13-314C10.5
ENSG00000267239.1	lnc-PTPN2-3	-4.679775126	0.780238476	-6.003331952	1.34592E-06	0.003164373	5.024150495	RP11-794M8.1
ENSG00000259999.1	lnc-BCAR1-1	4.637458356	2.560182004	5.969768037	1.47885E-06	0.003164373	5.09070952	RP11-252K23.1
ENSG00000263603.1	lnc-ATAD5-2	4.911055198	2.660170693	5.773550579	2.56818E-06	0.004396218	4.61840665	CTD-2349P21.5
ENSG00000263050.1	lnc-SMG6-2	5.141973236	4.175964552	5.617287588	3.99124E-06	0.005569173	4.173139114	RP11-667K14.3
ENSG00000245164.6	LINC00861	-3.636475423	7.569837542	-5.545622871	4.88726E-06	0.005569173	4.058452038	LINC00861
ENSG00000253532.1	lnc-OSR2-2	4.903044063	3.559107054	5.51421332	5.34119E-06	0.005569173	3.801701012	CTD-2340D6.1
ENSG00000235532.1	LINC00402	-4.258482048	5.198993538	-5.481675931	5.85612E-06	0.005569173	3.927182148	LINC00402
ENSG00000198685.3	LINC01565	4.311160072	1.859317249	5.204681314	1.28313E-05	0.009804976	3.156579163	LINC01565
ENSG00000249667.1	LINC01259	-5.203618859	2.58881354	-5.153191488	1.48468E-05	0.009804976	3.009787549	LINC01259
ENSG00000228315.11	lnc-IGLL1-1	3.879119909	2.119259742	5.141354221	1.53532E-05	0.009804976	2.983421374	GUSBP11
ENSG00000249464.5	lnc-SPRY1-11	5.182439917	2.614524535	5.126646778	1.60065E-05	0.009804976	2.996016827	LINC01091
ENSG00000236753.5	MKLN1-AS	4.991332242	4.342386467	5.125951881	1.6038E-05	0.009804976	2.701563512	MKLN1-AS
ENSG00000258499.1	LINC02287	4.345368365	2.28438899	5.068811141	1.88564E-05	0.010759484	2.815977193	RP11-862G15.2
ENSG00000233355.6	CHRM3-AS2	-3.305853448	7.187867406	-4.941811062	2.70186E-05	0.014453269	2.427926726	CHRM3-AS2
ENSG00000237372.2	lnc-GADD45G-3	4.837842786	2.336330225	4.879263053	3.22508E-05	0.016237328	2.352160441	UNQ6494
ENSG00000253619.1	lnc-MTBP-2	3.858831608	2.445657706	4.824981982	3.76025E-05	0.017879982	2.191188859	RP11-369K17.1
ENSG00000263724.1	DLGAP1-AS3	-4.594500961	1.606209771	-4.80093371	4.02478E-05	0.018130572	2.091443118	DLGAP1-AS3
ENSG00000254983.1	lnc-PARVA-7	4.10687825	2.753363318	4.762491981	4.48662E-05	0.018142251	2.038831786	RP11-573E11.2
ENSG00000241657.1	lnc-MGAM2-1	-4.854386228	2.159875088	-4.754714227	4.58628E-05	0.018142251	1.983207197	TRBV11-2
ENSG00000250390.2	lnc-CEP57-2	-4.693453609	1.431276471	-4.748820348	4.66327E-05	0.018142251	1.96654407	RP11-338H14.1
ENSG00000249631.5	lnc-DRD5-10	4.119021252	1.167586555	4.730451656	4.91156E-05	0.018277395	1.956815852	RP11-281P23.2
ENSG00000276445.1	lnc-DYRK1B-2	3.967872261	0.930648641	4.688163782	5.53422E-05	0.019364345	1.848632528	LLNLR-268E12.1
ENSG00000203279.3	lnc-CTSV-5	3.643886709	1.167302625	4.680440488	5.65614E-05	0.019364345	1.825421167	RP11-498P14.5
ENSG00000270190.1	lnc-ANAPC1-5	3.751176251	1.809993757	4.599848105	7.09876E-05	0.023359792	1.625417306	RP11-803D5.4
ENSG00000260988.1	lnc-SH2D7-1	4.150567101	1.451055941	4.584279351	7.4169E-05	0.023359792	1.592346198	RP11-285A1.1
ENSG00000243179.1	LINC01191	4.991728666	2.911662543	4.57366154	7.64195E-05	0.023359792	1.580734413	AC110769.3
ENSG00000203364.2	lnc-CARD19-4	4.388783214	1.853266272	4.534044083	8.54303E-05	0.025213712	1.470846582	RP11-370F5.4
ENSG00000242082.1	SLC5A4-AS1	-4.339549475	0.686476165	-4.467451103	0.000103008	0.029388129	1.267387261	RP1-90G24.10
ENSG00000231842.1	lnc-TRMT11-2	3.651812504	2.256386076	4.44779162	0.000108851	0.030053293	1.245520016	RP11-527F13.1
ENSG00000257953.1	lnc-TSFM-1	3.633224303	1.198425691	4.425660128	0.000115821	0.030978523	1.190603745	RP11-620J15.1
ENSG00000260209.1	lnc-PUS3-1	3.252912968	0.623389801	4.353813964	0.000141635	0.036654679	1.011411167	RP11-680F20.10
ENSG00000273348.1	lnc-CTAGE1-6	-3.964737662	0.349529447	-4.341908618	0.00014643	0.036654679	0.949630862	RP11-535A5.1
ENSG00000271959.1	/	3.589472202	1.817604842	4.324882373	0.000153568	0.036654679	0.940421112	CTD-3064M3.7
ENSG00000264745.1	TTC39C-AS1	-4.127363067	1.747777487	-4.323474914	0.000154173	0.036654679	0.894637493	TTC39C-AS1
ENSG00000275383.1	lnc-ZFP90-4	3.60668482	1.255924237	4.304837144	0.000162412	0.03756996	0.890828916	RP11-615I2.6
ENSG00000255801.1	lnc-CLEC4E-1	5.390954926	3.872496476	4.286828471	0.000170786	0.038467296	0.855904992	RP11-561P12.5
ENSG00000235576.1	LINC01871	-4.360222142	2.586999356	-4.271803256	0.000178097	0.039085463	0.789402556	AC092580.4
ENSG00000254739.1	lnc-LRRC56-1	3.351207604	1.326927296	4.236657658	0.000196426	0.041199474	0.721894246	RP13-46H24.1
ENSG00000266835.5	GAPLINC	3.696360907	1.189602401	4.234959439	0.000197357	0.041199474	0.718673497	GAPLINC
ENSG00000224652.1	LINC00885	3.969101125	1.72865799	4.218762219	0.00020646	0.042073699	0.679731277	LINC00885
ENSG00000246223.8	LINC01550	-3.319209735	5.395895698	-4.167248767	0.000238252	0.04709742	0.511118994	LINC01550
ENSG00000223662.1	/	4.2379911	3.145812525	4.161451559	0.000242118	0.04709742	0.540453837	SAMSN1-AS1
ENSG00000235785.1	lnc-CCNK-1	-4.054068331	2.020682054	-4.15204783	0.000248521	0.04726872	0.482676947	AL109767.1
ENSG00000225329.3	LHFPL3-AS2	3.340523198	1.93405681	4.114064119	0.000276125	0.049834861	0.420442043	LHFPL3-AS2
ENSG00000269600.1	lnc-MZF1-1	-4.235130564	3.070294095	-4.113021268	0.000276924	0.049834861	0.421050615	AC016629.3
ENSG00000228022.5	HCG20	4.111946138	2.055767261	4.109703495	0.00027948	0.049834861	0.411964638	HCG20

Table S5. Fold changes for differentially expressed lncRNAs on qPCR between PN and JMML subgroups.

abbreviations: lncRNA = long non-coding RNA; qPCR = quantitative reverse transcriptase PCR; FC = fold change; 5N = Quintuple negative JMML samples.

	Fold Change						
**lncRNA**	**PN**	**JMML**	**KRAS**	**NRAS**	**PTPN11**	**NF1**	**5N**
lnc-THADA-4 transcript 3and4	1	3.91	0.88	2.60	5.73	6.82	6.55
lnc-THADA-4 transcript 1	1	7.68	1.65	4.63	12.16	11.04	14.26
lnc-ACOT9-1	0	2.66	3.49	1.75	2.04	5.87	0.88
NRIR transcript 1	1	5.83	1.60	6.68	4.59	15.11	2.81
NRIR transcript 2	1	4.83	2.14	5.47	3.64	12.15	2.07
lnc-ACSL1-1 transcript 3	1	2.69	2.90	2.24	2.36	4.71	2.02
lnc-ACSL1-1 transcript 4	1	11.49	8.90	20.17	2.93	25.12	11.32
LINC02217	1	6.37	4.70	9.68	3.29	1.08	19.99
MEG3	1	5.23	5.37	5.60	7.70	0.63	1.37
lnc-EGR3-1	1	12.34	7.24	20.37	9.02	18.01	8.48
MIR222HG	1	5.15	4.98	8.66	3.16	3.56	4.76
lnc-RUNX3-4	0	0.010	0.004	0.013	0.012	0.004	0.015
LINC01270	0	0.778	0.673	1.803	0.721	0.000	0.473
GASAL1	0	0.015	0.000	0.009	0.016	0.023	/
lnc-SPOPL-18	0	1.938	3.015	1.521	2.507	0.000	0.000
LINC02218	1	3.37	1.50	6.46	1.79	6.82	/
lnc-FCAR-1	1	2.95	2.49	2.96	2.96	2.33	4.78
lnc-ANTXR2-1	1	3.24	1.57	3.61	4.18	5.05	1.18
SH3RF3-AS1	1	4.13	4.83	3.78	4.68	4.26	1.66
lnc-CYP4F22-4	1	3.72	3.35	3.79	2.79	2.77	9.39
MKLN1-AS	1	1.98	1.45	1.81	1.33	5.91	1.49

Table S6. Haematopoietic cell lines with expression of lncRNA based on CCLE RNAseq data and validated by qPCR

abbreviations: lncRNA = long non-coding RNA; qPCR = quantitative reverse transcriptase PCR; NRQ = normalized relative quantity; CCLE = the cancer cell line encyclopedia; RNAseq = RNA sequencing; TPM = transcripts per million.

	lnc-THADA-4		lnc-ACOT9-1		NRIR		lnc-ACSL1-1		lnc-EGR3-1		MIR222HG		LINC02217		lnc-CYP4F22-4		lnc-RUNX3-4		MEG3	
Haematopoietic cell lines	RNAseq TPM	qPCR (NRQ)	RNAseq TPM	qPCR (NRQ)	RNAseq TPM	qPCR (NRQ)	RNAseq TPM	qPCR (NRQ)	RNAseq TPM	qPCR (NRQ)	RNAseq TPM	qPCR (NRQ)	RNAseq TPM	qPCR (NRQ)	RNAseq TPM	qPCR (NRQ)	RNAseq TPM	qPCR (NRQ)	RNAseq TPM	qPCR (NRQ)
DEL, anaplastic large cell lymphoma	/	0.0013	6	0.0740	0.7000	0.0178	863	1	/	0.0000	/	0.0495	0.1000	0.0288	0.3000	0.1533	15	0.0000	0.1000	0.0000
Daudi, Burkitts lymphoma	/	0.0079	7	0.1875	9	0.0258	0.1000	0.0000	/	0.0000	0.2000	0.0005	/	0.0000	10	0.9501	/	0.0000	/	0.0001
HL-60, adult acute myeloid leukemia	/	0.0031	/	0.4386	0.1000	0.0003	0.1000	0.0000	/	0.0000	0.1000	0.0000	0.5000	0.0000	0.2000	0.0945	3	0.0000	/	0.0000
JURKAT, childhood T acute lymphoblastic leukemia	0.3000	0.0296	3	0.0767	1	0.0404	/	0.0000	/	0.0000	1	0.0000	/	0.0000	0.5000	0.1451	/	0.0000	/	0.0001
JVM-2, Mantle cell lymphoma	/	0.0003	7	0.4137	0.2000	0.0212	9	0.0018	0.7000	0.0312	0.4000	0.1110	0.3000	0.2753	2	0.1850	4	1	3	0.0136
K-562, blast phase chronic myelogenous leukemia, BCR-ABL1 positive	/	0.0000	5	1.0000	0.6000	1	14	0.0132	/	1	/	0.0600	/	0.0000	1	0.3399	/	0.0000	4	1
KARPAS-422, diffuse large B-cell lymphoma	/	0.0007	17	0.7439	58	0.2664	/	0.0000	/	0.0000	/	0.0000	/	0.0000	1	0.1053	/	0.0000	/	0.0000
KASUMI-1, childhood acute myeloid leukemia with maturation	/	0.0005	/	0.0000	0.3000	0.0146	/	0.0000	/	0.0000	0.7000	0.1162	0.2000	0.0502	1	0.0529	/	0.0000	/	0.0001
Loucy, adult T acute lymphoblastic leukemia	0.4000	0.0011	/	0.0000	10	0.1851	0.1000	0.0000	/	0.0712	4	0.1246	/	0.0000	2	0.0796	0.1000	0.0000	0.1000	0.0000
MONO-MAC-6, adult acute monocytic leukemia	/	0.0028	0.3000	0.0000	1	0.0353	7	0.0077	/	0.0000	0.4000	0.4943	0.3000	0.1814	0.5000	0.1904	/	0.0000	/	0.0000
MV-4-11, childhood acute monocytic leukemia	0.2000	0.0012	0.1000	0.0000	0.3000	0.0363	0.1000	0.0000	/	0.0007	0.0000	0.1295	0.4000	0.5189	1	0.0308	0.6000	0.0000	/	0.0000
OCI-AML3, adult acute myeloid leukemia	/	0.0024	/	0.0000	0.3000	0.0016	0.1000	0.0013	0.1000	0.0000	0.0000	0.1489	0.9000	1	2	0.1823	/	0.0000	0.3000	0.0000
PEER, childhood T acute lymphoblastic leukemia	14	1	1	0.1582	3	0.0791	/	0.0000	/	0.0000	3	0.0014	/	0.0000	0.7000	0.0723	/	0.0000	/	0.0000
THP-1, childhood acute monocytic leukemia	/	0.1730	0.1000	0.0000	8	0.7190	0.1000	0.0000	/	0.0000	0.2000	1	0.2000	0.0000	0.5000	1	0.1000	0.0000	0.1000	0.0005

Supplementary Figures

Supplementary Figure 1: qPCR validation of the most significant DEGs discovered by RNA sequencing in the validation cohort only. qPCR confirmed upregulation of lncRNA expression of 13 lncRNAs (9 significant ($P \leq 0.05$) and 4 near significant ($P < 0.1$) in JMML compared to PN controls. One-sided P-values were calculated by the Mann-Whitney U test. Median values and quartiles are shown by horizontal lines. The vertical axis (y-axis) corresponds to the calibrated normalized relative quantities (CNRQ). *TBP*, *HPRT1* and *GAPDH* were used as internal reference genes. *Abbreviations: qPCR = quantitative reverse transcriptase PCR, JMML = juvenile myelomonocytic leukemia, PN = pediatric normal, lncRNA = long non-coding RNA, CNRQ = calibrated normalized relative quantity.*

Supplementary Figure 2: Correlation between lncRNA expression on RNA sequencing and qPCR for validated lncRNAs. Pearson correlation coefficients were generated with R bioconductor. For two lncRNAs (*lnc-SPOPL-18* and *LINC01270*) there was insufficient data to generate sufficiently reliable results. *Abbreviations: qPCR = quantitative reverse transcriptase PCR, lncRNA = long non-coding RNA.*

Supplementary Figure 3: Comparison of molecular subgroups. qPCR expression data of 19 lncRNA transcripts in JMML of 5 molecular subtypes (*KRAS*, *NRAS*, *PTPN11* and *NF1* mutated and quintuple negative [5N]) and in PN were used to test differential expression of all molecular subgroups compared with PNBMs and with each other. The vertical axis (y-axis) corresponds to the calibrated normalized relative quantities (CNRQ). *TBP*, *HPRT1* and *GAPDH* were used as internal reference genes. Two-sided P-values were calculated by the Kruskall-Wallis test. Only P values ≤ 0.1 are shown for the corresponding pairwise comparisons. *Abbreviations: qPCR = quantitative reverse transcriptase PCR, JMML = juvenile myelomonocytic leukemia, PN = pediatric normal, CNRQ = calibrated normalized relative quantity.*

Supplementary Figure 4: The level of molecular expression of the lncRNA of interest in a selection of hematopoietic cell lines was evaluated with qPCR. Hematopoietic cell lines were chosen based on lncRNA of interest expression in RNA sequencing data from the Cancer Cell Line Encyclopedia.

Molecular expression was relative to the average expression of three different housekeeping genes (*TBP*, *HPRT1* and *GAPDH*) and rescaled against the cell line with highest expression. *Abbreviations: qPCR = quantitative reverse transcriptase PCR, lncRNA = long non-coding RNA, NRQ = normalized relative quantity.*

Supplementary Figure 5: Level of molecular knockdown in hematopoietic cell lines using LNA GapmeRs. Efficiency of lncRNA knockdown using a minimum of 4 different LNA GapmeRs against the lncRNA of interest after 24h incubation with 5 µM concentration. Hematopoietic cell lines were chosen based on lncRNA of interest expression in RNA sequencing data from the Cancer Cell Line Encyclopedia and qPCR validations. The level of molecular expression was normalized against the average expression of three different housekeeping genes (*TBP*, *HPRT1* and *GAPDH*) and rescaled against the mock-control (TE-buffer). Knockdown was considered successful if down regulation of ≥ 70% could be observed. *Abbreviations: qPCR = quantitative reverse transcriptase PCR, lncRNA = long non-coding RNA, LNA = locked nucleic acid, µM = micro molar, NRQ = normalized relative quantity.*

Supplementary Figure 1

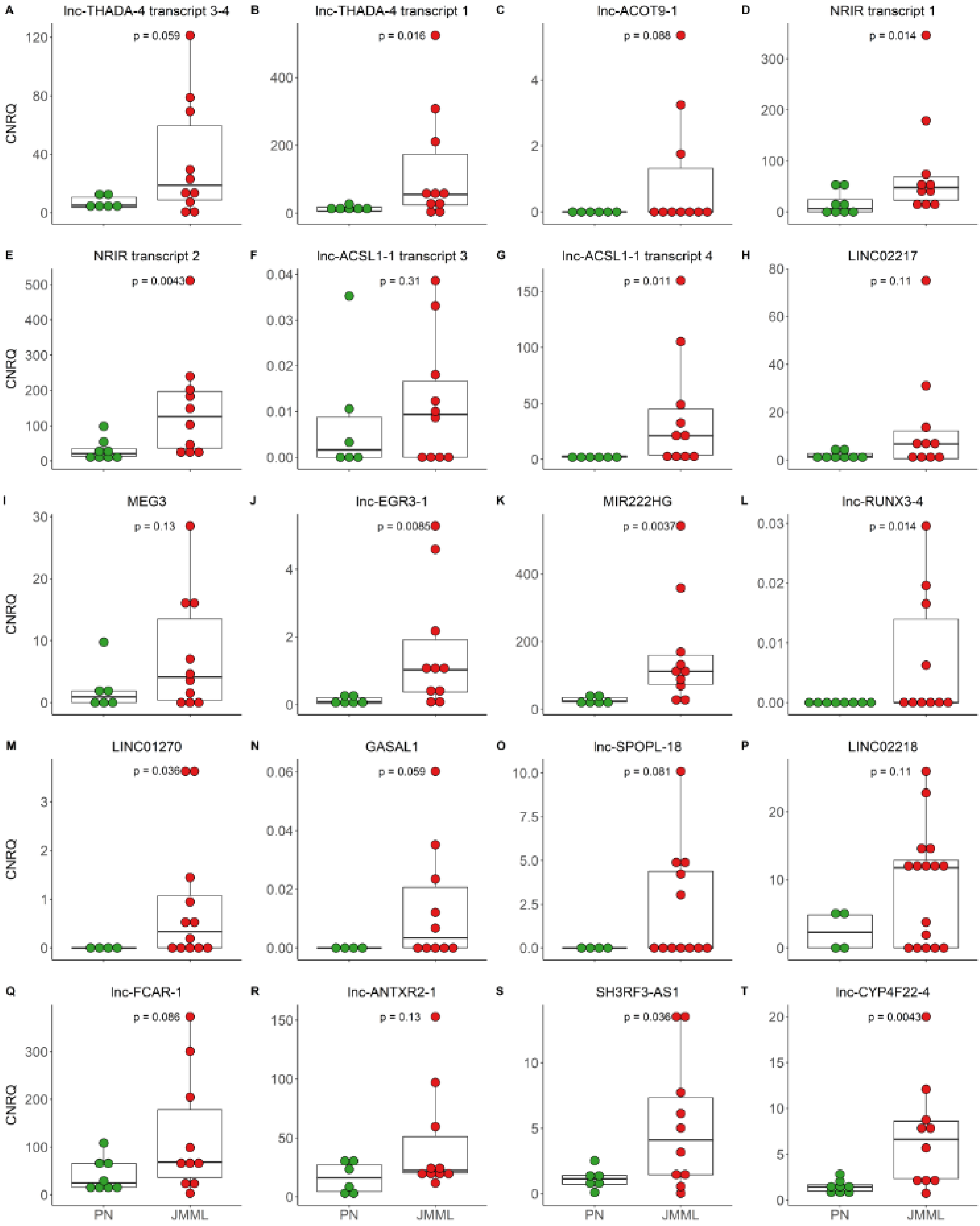

Supplementary Figure 2:

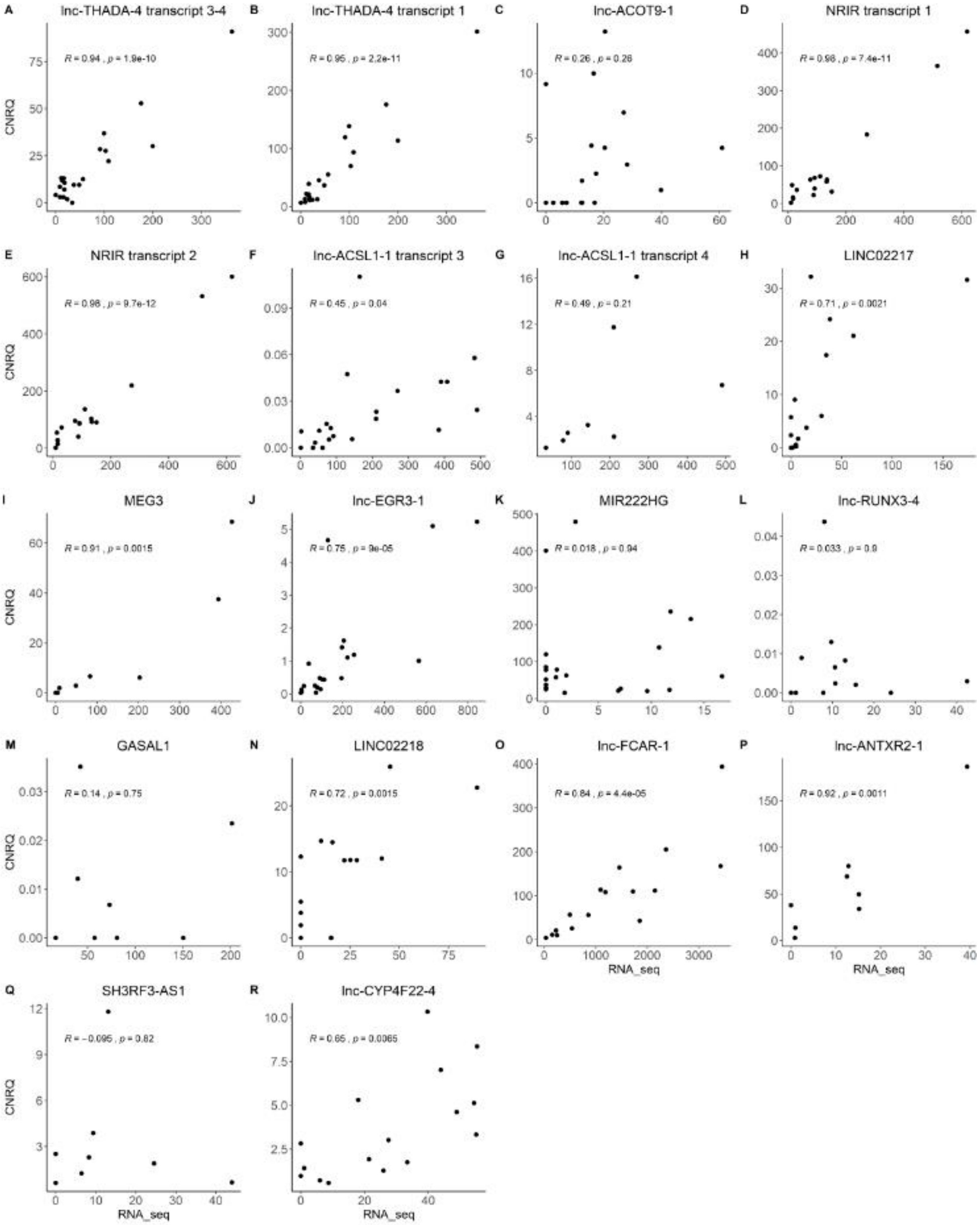

Supplementary Figure 3

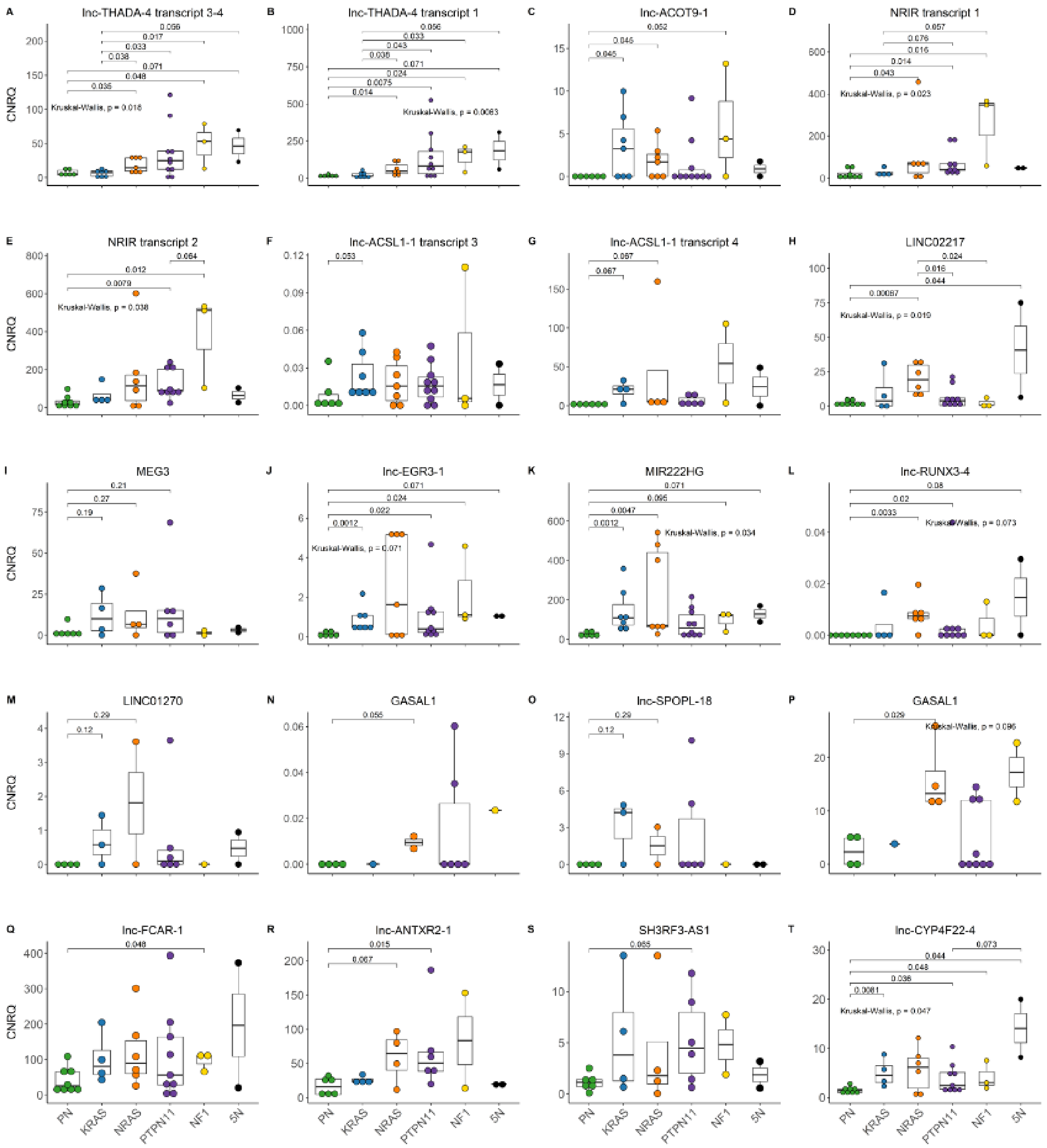

Supplementary Figure 4

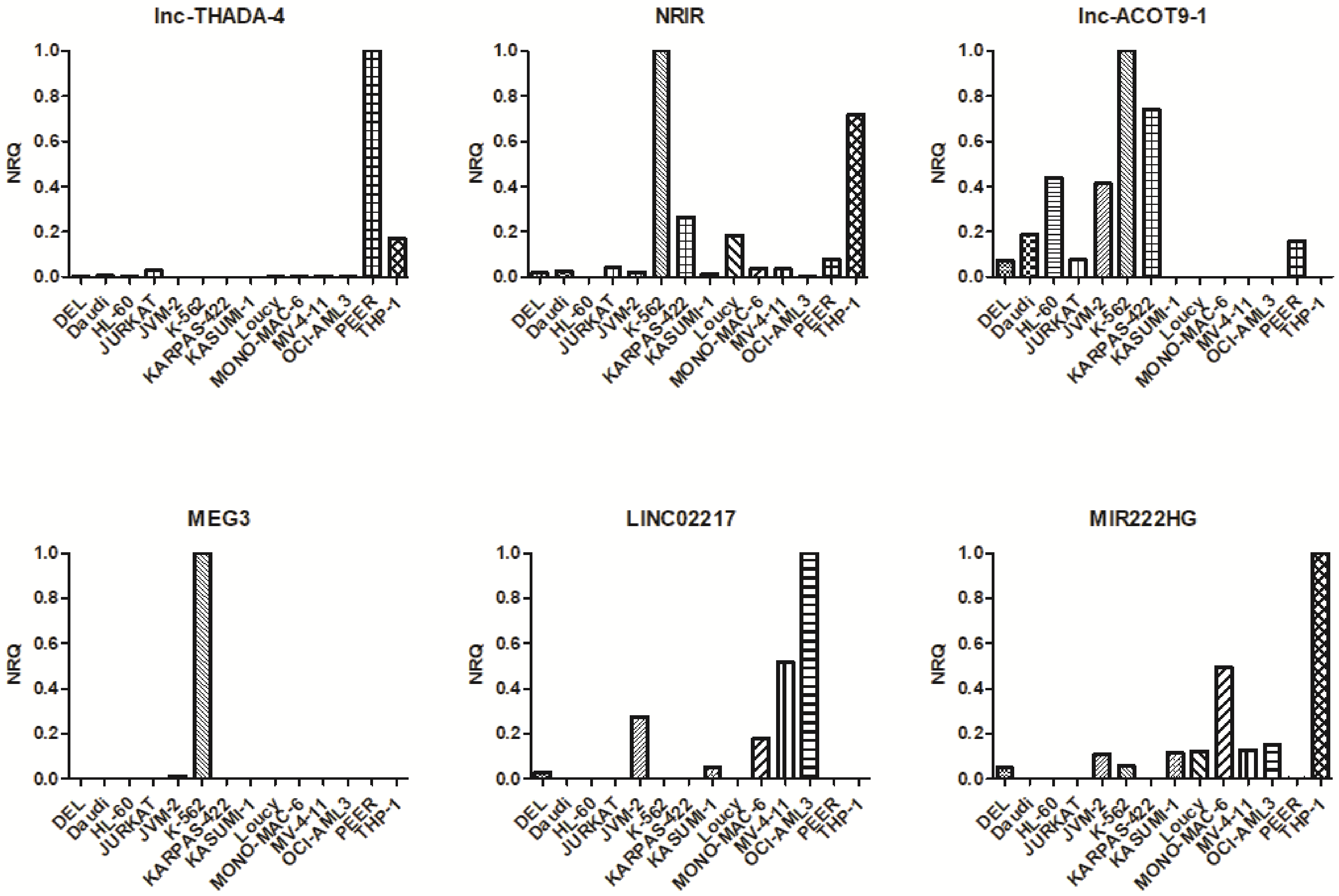

Supplementary Figure 5

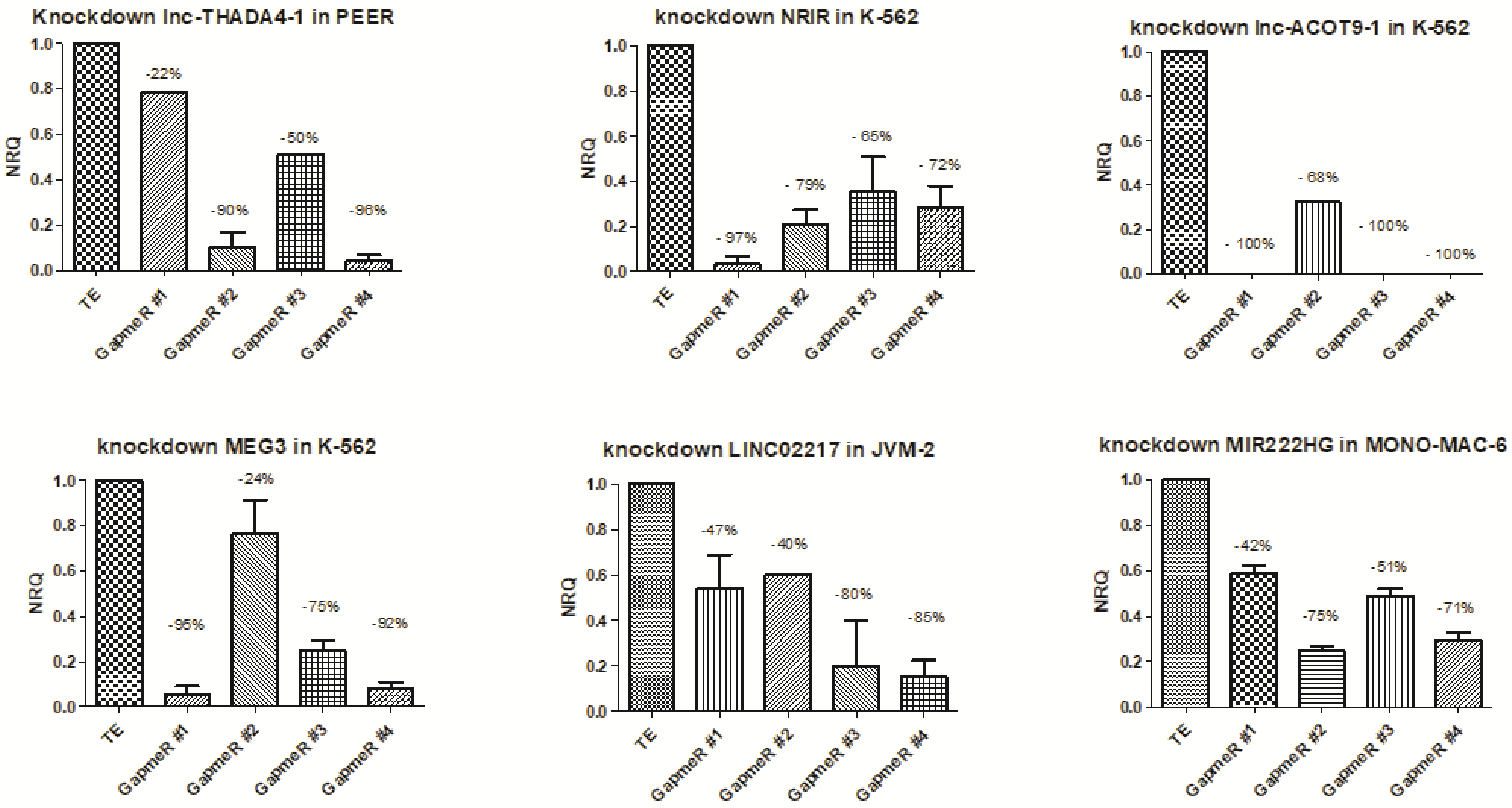

Supplement: Supplementary file 1 — Supplementary Information [file 41598_2021_82509_MOESM1_ESM.pdf]
